# PBRM1 Deficiency in Microglia Leads to Excessive Neurogenesis and Autism‐Like Behaviors via the CCL5‐CCR1/5‐β‐catenin Signaling Axis

**DOI:** 10.1002/advs.78086

**Published:** 2026-10-18

**Authors:** Yang‐Yang Duan, Lin‐Fei Jiao, Pei‐Pei Liu, Chun‐Hui Duan, Xiao Li, Zi‐Han Zhang, Yue‐Qi Zhang, Xin‐Li Ouyang, Hong‐Zhen Du, Sha Liu, Yi Tang, Zhao‐Qian Teng, Chang‐Mei Liu

**Affiliations:** ^1^ State Key Laboratory of Organ Regeneration and Reconstruction Institute of Zoology Chinese Academy of Sciences Beijing China; ^2^ University of Chinese Academy of Sciences Beijing China; ^3^ Institute for Stem Cell and Regeneration Chinese Academy of Sciences Beijing China; ^4^ Center for Brain & Disease Research VIB‐KU Leuven Leuven Belgium; ^5^ Department of Neurology Innovation Center for Neurological Disorders Xuanwu Hospital Capital Medical University Beijing China

**Keywords:** autism spectrum disorder, CCL5, microglial homeostasis, neurogenesis, PBRM1

## Abstract

The prevalence of autism spectrum disorder (ASD), a heterogeneous neurodevelopmental disorder with limited effective therapeutic drugs, has been rising worldwide. Missense mutations in Polybromo 1 (*Pbrm1*), a core component of the SWI/SNF (switch/sucrose non‐fermentable) chromatin remodeling complex, have been identified as a risk factor for ASD; however, whether PBRM1 loss of function contributes to the pathogenesis remains elusive. Here we provide direct evidence that microglial PBRM1 deficiency leads to altered microglial clusters, excessive neurogenesis, and ASD‐like behaviors in mice. Integrated molecular analyses reveal that PBRM1 directly binds to the *Ccl5* promoter and represses the expression of CCL5. Elevated CCL5 promotes the proliferation and neuronal differentiation of neural stem/progenitor cells (NSPCs) via the CCR1/5‐β‐catenin signaling pathway. Importantly, pharmacological blockade of CCR1/5 ameliorates behavioral deficits in *Pbrm1* cKO mice. Together, these findings identify a previously unrecognized microglia–NSPC communication mechanism linking PBRM1 dysfunction to aberrant neurodevelopment and suggest the CCL5–CCR1/5 axis as a potential therapeutic target for ASD.

## Graphical abstract

Microglial PBRM1 deficiency drives excessive hippocampal neurogenesis by enhancing CCL5–CCR1/5 signaling, leading to autism‐like behaviors. A pro‐inflammatory IL1α^+^/TNFα^+^ microglial subset is identified as a key cellular contributor to this process. PBRM1 represses *Ccl5* expression at the transcriptional level. Pharmacological inhibition of CCR1/5 alleviates neurogenic and behavioral abnormalities, highlighting a potential therapeutic strategy for autism spectrum disorder.

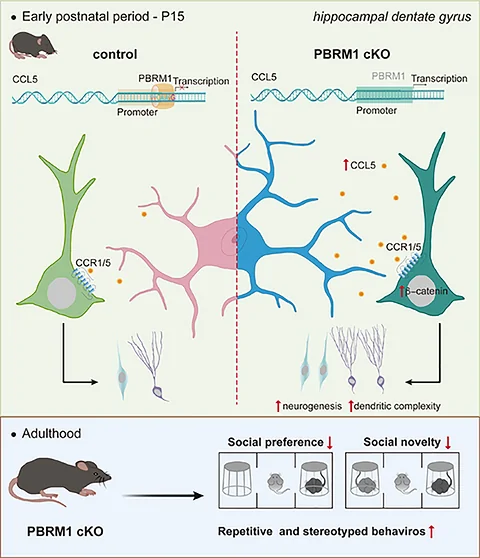

## 1 Introduction

Autism spectrum disorder (ASD) is a heterogeneous neurodevelopmental disorder characterized by repetitive behaviors and difficulties in social communication [1, 2, 3]. Increasing evidence indicates that these behavioral abnormalities arise from dysfunctional neural circuits spanning multiple brain regions, including the prefrontal cortex, striatum, amygdala, hippocampus, and cerebellum, which collectively contribute to social, emotional, and cognitive abnormalities [4]. Genetic studies have revealed that variants in hundreds of genes contribute to approximately 10%–30% of ASD cases [5, 6, 7, 8]. Notably, genes encoding components of the BAF (mammalian SWI/SNF) chromatin‐remodeling complex are among the recurrently implicated risk loci [9, 10, 11]. For instance, ACTL6B, a neuron‐specific BAF subunit, is the most significantly mutated gene identified in the Simons Recessive Autism Cohort [12]. Loss of ACTL6B impairs neuron‐specific chromatin repression and causes ASD‐like phenotypes in both human brain organoids and experimental animal models [12].

Rare missense variants in *PBRM1* (also known as *BAF180*) have been reported in individuals with sporadic ASD [9]. In addition, a population‐based study in Taiwan identified a significant association between the *PBRM1* rs2251219 polymorphism and susceptibility to major depressive disorder [13]. Beyond its genetic association with neuropsychiatric disorders, PBRM1 functions as a context‐dependent chromatin regulator that preserves the regenerative capacity of epithelial progenitor cells while promoting transcriptional activation during cellular differentiation [14]. These observations suggest that PBRM1 may serve as an important regulator of lineage specification and developmental progression. Consistent with this possibility, PBRM1 is broadly expressed throughout oligodendroglial development during both prenatal and postnatal stages of the central nervous system (CNS) [15]. Genetic ablation of *Pbrm1* primarily affects the transition from oligodendrocyte progenitor cells to pre‐myelinating oligodendrocytes, ultimately reducing the production of mature myelinating oligodendrocytes [15]. Nevertheless, despite its widespread expression in the brain, the cell type‐specific functions of PBRM1 and the molecular mechanisms underlying its roles in neurodevelopment and neurological disorders remain poorly understood.

Mounting evidence indicates that microglia, the brain's resident immune cells, regulate neurodevelopment through synaptic pruning and the regulation of neurogenesis and are also implicated in ASD pathogenesis [16, 17]. Notably, the hippocampus is the principal region of postnatal neurogenesis and exhibits structural and functional abnormalities in ASD. In this study, we generated microglia‐specific *Pbrm1* conditional knockout mice to investigate the impact of loss of microglial PBRM1 in vivo. We show that loss of microglial PBRM1 leads to altered microglial clusters, excessive hippocampal neurogenesis, increased dendritic spine density, and ultimately results in ASD‐like behavioral abnormalities. Integrative multi‐omics analyses combined with ChIP and luciferase reporter assays further revealed that PBRM1 binds to the *Ccl5* promoter and represses its transcription. Elevated CCL5 subsequently promotes the proliferation and neuronal differentiation of neural stem/progenitor cells (NSPCs) via the CCR1/5‐β‐catenin signaling pathway. Importantly, pharmacological inhibition of CCR15 substantially ameliorates the deficits associated with microglial PBRM1 deficiency. Collectively, our findings highlight a critical role of microglial PBRM1 in brain development and suggest that the dysregulated PBRM1‐CCL5‐CCR1/5 axis may contribute to the pathogenesis of ASD.

## 2 Results

### 2.1 Loss of Microglial PBRM1 Leads to Autism‐Like Behaviors in Mice

To explore the functional role of PBRM1 in microglia, we generated a microglia‐specific conditional *Pbrm1* knockout mouse line by crossing *Pbrm1 ^flox/flox^* and *Cx3cr1‐Cre* mice to produce *Cx3cr1‐Cre^+/−^*;*Pbrm1^flox/flox^* mice (*Pbrm1* cKO). Immunostaining and Western blotting analyses indicated that *Pbrm1* is specifically ablated in microglia in *Pbrm1* cKO mice compared to *Cx3cr1‐Cre^−/−^*;*Pbrm1 ^flox/flox^* control littermates (*Pbrm1* WT) (Figure S1).

Given the association of PBRM1 with neurodevelopmental disorders, we then assessed whether the loss of microglial PBRM1 leads to any behavioral abnormalities relevant to these conditions, including anxiety‐like and social behaviors. In the open field test (OFT), although there were no significant differences in total locomotor activity between WT and *Pbrm1* cKO mice (Figure 1A,B), cKO mice exhibited significantly fewer entries and less time spent in the center of the open field (Figure 1A,B). Similarly, in the elevated‐plus maze test (EPM), *Pbrm1* cKO mice spent less time in the open arms, further suggesting anxiety‐like behavior in *Pbrm1* cKO mice (Figure 1C,D).

**FIGURE 1:**
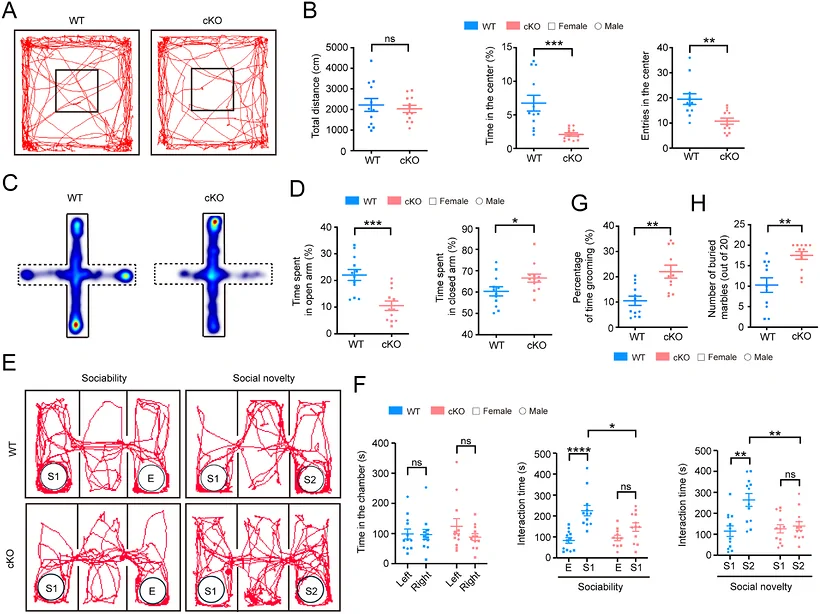
*Pbrm1* cKO mice exhibit autism‐like behavior and anxiety‐like behaviors. (A,B) Representative trajectories (A) and quantifications (B) of total distance moved, time spent, and entries in the center zone of *Pbrm1* WT and cKO mice in a 10 min test period of the open field test. The black square represents the center zone of the field. Mean ± SEM. ns, no significant, ^**^ *p*< 0.01, ^***^ *p*< 0.001 by Student's *t*‐test. n = 12 mice per group. Males (circles) and females (squares) are indicated. (C,D) Representative trajectories (C), quantifications (D) of time spent in open arms and time spent in closed arms in the elevated plus maze test. The *Pbrm1* cKO mice spent less time in the open arms compared with WT mice. Mean ± SEM. ^*^ *p*< 0.05, ^***^ *p*< 0.001 by Student's *t*‐test. n = 12 mice per group. Males (circles) and females (squares) are indicated. (E,F) Representative trajectories (E) and quantifications (F) of time spent in the chambers, interaction time from the Stranger 1‐Empty and Stranger 1–Stranger 2 trials in the three‐chamber test. The *Pbrm1* cKO mice showed no preference for sociability. S1, Stranger 1; E, Empty; S2, Stranger 2. Mean ± SEM. ns, no significant; ^*^ *p*< 0.05; ^**^ *p*< 0.01; ^****^ *p*< 0.0001 by Two‐way ANOVA followed by Šídák's multiple comparisons test. n = 11–12 mice per group. Males (circles) and females (squares) are indicated. (G) Self‐grooming test showing an increased total time spent grooming in *Pbrm1* cKO mice than in WT mice. Mean ± SEM. ^**^ *p*< 0.01 by Student's *t*‐test. n = 11–12 mice per group. Males (circles) and females (squares) are indicated. (H) Quantification of the marble burying score per group. Data showed that the *Pbrm1* cKO mice buried a greater number of marbles. Mean ± SEM. ^**^ *p*< 0.01 by Mann–Whitney test. n = 11–12 mice per group. Males (circles) and females (squares) are indicated. See also Figures S1 and S2.

To further assess the impact of microglial PBRM1 deficiency on social behavior, we performed the three‐chamber social interaction test and found that *Pbrm1* cKO mice exhibited reduced social interactions (Figure 1E,F). Similarly, the marble burying and grooming tests showed that *Pbrm1* cKO mice exhibited a significantly greater number of marble burying bouts, as well as increased total time spent in self‐grooming (Figure 1G,H). Collectively, these data indicate that microglial PBRM1 deficiency leads to anxiety and autism‐like behaviors in mice.

To assess whether loss of PBRM1 in microglia impairs learning and memory, we performed the Morris water maze, Y‐maze, and novel object recognition (NOR) tests. In the Morris water maze test, there was no significant difference in escape latency between WT and cKO mice during the training phase (Figure S2A). In the probe trial, cKO mice exhibited no significant differences in the number of target platform crossings or the time spent in the target quadrant compared with WT mice (Figure S2B,C). Similarly, cKO mice exhibited no significant difference in spontaneous alternation performance (Figure S2D). In the NOR test, cKO mice exhibited a significantly decreased object recognition index, indicating impaired recognition memory (Figure S2E). Collectively, these results indicate that PBRM1 deficiency does not significantly affect spatial learning and working memory but impairs recognition memory.

### 2.2 Modified Hippocampal Neurogenic Niche in *Pbrm1* cKO Mice

Given the social and emotional deficits observed in *Pbrm1* cKO mice and the contribution of newborn hippocampal neurons to social behavior [18, 19, 20, 21], we examined whether the loss of microglial PBRM1 affected hippocampal neurogenesis. First, we isolated hippocampal tissues from P15 WT and *Pbrm1* cKO mice and performed single‐cell RNA sequencing (scRNA‐seq) using the 10× Genomics platform. Unsupervised clustering analysis and uniform manifold approximation and projection (UMAP) visualization identified eight major cell types, including microglia (MG), neurons, astrocytes (AST), NSCs, oligodendrocytes (OLIG), endothelial cells, ependymal cells, and less abundant cell types (approximately 1%) (Figure S3), based on transcriptomic differentially expressed genes (DEGs) and previously reported cell‐type‐specific markers [22, 23, 24]. Next, we used the CellChat toolkit to infer and analyze signaling network communication among microglia, neurons, and NSCs based on ligand‐receptor expression patterns. We found that both the number and overall strength of predicted communications were increased in cKO mice compared to WT mice (Figure S4A), suggesting that microglial *Pbrm1* deficiency may activate additional signaling pathways or enhance existing signaling activity. Intriguingly, the inferred outgoing and incoming signals between microglia and NSCs were preferentially increased relative to microglia‐neuron signaling (Figure S4B), indicating a stronger functional communication between microglia and NSPCs.

To further assess whether microglia are physically positioned to communicate with NSPCs, we conducted IBA1 and SOX2 immunostaining in *Pbrm1* cKO; Nestin‐GFP mouse brains. We observed a significantly increased ratio of microglia to NSPCs in *Pbrm1* cKO mice compared to control mice (Figure S4C), a spatial association consistent with enhanced crosstalk between microglia and NSPCs. We then analyzed the single‐cell transcriptomic data of hippocampal NSCs, neurons, and astrocytes to investigate downstream pathways potentially affected by microglial *Pbrm1* deficiency. Gene Ontology (GO) enrichment analysis revealed that NSCs in *Pbrm1* cKO mice showed upregulation of pathways related to proliferation and neuronal differentiation (Figure S4D). Neurons exhibited upregulation of synaptic and dendritic development pathways (Figure S4E), and astrocytes displayed enrichment for processes supporting neurogenesis (Figure S4F). These results suggest that microglial *Pbrm1* deficiency may influence the proliferation and differentiation of NSPCs.

### 2.3 Excessive Neurogenesis in *Pbrm1* cKO Mice

Given that neurogenesis primarily occurs in the hippocampal dentate gyrus (DG) and the subventricular zone (SVZ) in the postnatal stage, we investigated whether *Pbrm1* cKO in microglia increased the proliferation and differentiation of NSPCs in the hippocampus and SVZ. By applying bromodeoxyuridine (BrdU) pulse labeling in cKO and WT mice at P15 and P56, we detected a significantly higher number of BrdU^+^SOX2^+^Nestin‐GFP^+^ and BrdU^+^ cells in the DG of cKO mice (Figure 2A,B and Figure S5A,B). Meanwhile, the number of DCX^+^BrdU^+^ newborn neurons was also significantly increased in the hippocampus of *Pbrm1* cKO mice (Figure 2C,D and Figure S5A,B). Additionally, cKO mice showed a significantly higher number of NeuN^+^BrdU^+^ mature neurons (Figure 2C,D and Figure S5A,B). We also assessed neurogenesis in the SVZ and found that the pro‐neurogenic effects of microglial *Pbrm1* deletion were more pronounced in the hippocampus (Figure S5C,D). These data suggest that loss of microglial PBRM1 preferentially increases hippocampal neurogenesis.

**FIGURE 2:**
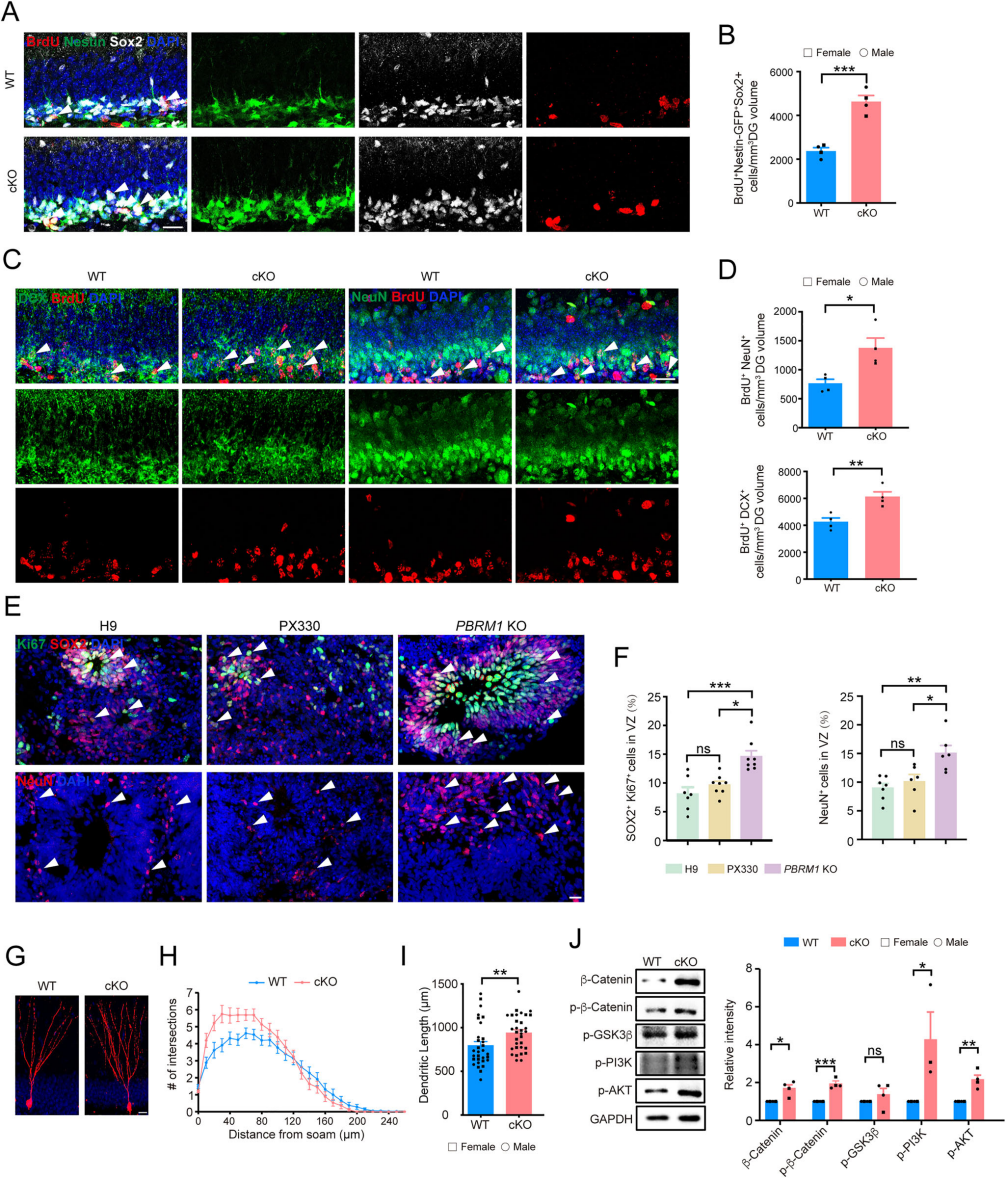
*Pbrm1* cKO microglia leads to excessive neurogenesis. (A) Confocal images of DAPI (blue), BrdU (red), Nestin‐GFP (green), and SOX2 (gray) immunostaining. Scale bars, 20 µm. (B) Quantification of the number of SOX2^+^Nestin‐GFP^+^BrdU^+^ NSPCs. The cKO mice had an increased number of SOX2^+^Nestin‐GFP^+^BrdU^+^ NSPCs. Mean ± SEM. ^***^ *p*< 0.001 by Student's *t*‐test. n = 4 mice per group. Males (circles) and females (squares) are indicated. (C) Confocal images of DAPI (blue), BrdU (red), and DCX (green) or NeuN (green) immunostaining. Scale bars, 20 µm. (D) Quantification analysis showed that cKO mice had an increase in BrdU^+^DCX^+^ immature neurons and an increased number of BrdU^+^NeuN^+^ neurons. Mean ± SEM. ^*^ *p*< 0.05, ^**^ *p*< 0.01 by Student's *t*‐test. n = 4 mice per group. Males (circles) and females (squares) are indicated. (E) Confocal images of DAPI (blue), Ki67 (green), SOX2 (red), or NeuN (red) immunostaining. Scale bars, 20 µm. (F) Quantification analysis showed that *PBRM1* KO microglia increased the ratio of Ki67^+^SOX2^+^ and NeuN^+^ cells. Microglia differentiated from H9 hESCs were added to organoids at day 18 (D18) and co‐cultured until D42 for analysis. Mean ± SEM. ns, no significant; ^*^ *p*< 0.05; ^**^ *p*< 0.01; ^***^ *p*< 0.001 by one‐way ANOVA followed by Tukey's multiple comparisons test or Kruskal–Wallis test followed by Dunn's multiple comparisons test, as appropriate. n = 7–8 batches, with 5–8 organoids analyzed per batch. (G–I) Representative images (G) and quantifications of intersections (H) and total dendritic length (I) of RFP‐labeled newborn neurons in WT and cKO mice at 28 days post‐injection. Scale bars, 5 µm. Newborn neurons in cKO mice had longer dendrites with complex branches. Mean ± SEM. ^**^ *p*<0.01 by Mann‐Whitney test. n = 31–32 neurons from 5 mice per group. Males (circles) and females (squares) are indicated. (J) Western blotting showed the expression levels of β‐catenin, p‐β‐catenin, p‐GSK3β, p‐AKT, and p‐PI3K in the hippocampus of WT and cKO mice. Mean ± SEM. ns, no significant, ^*^ *p*< 0.05, ^**^ *p*< 0.01, ^***^ *p*< 0.001 by Student's *t*‐test. n = 3–4 mice per group. Males (circles) and females (squares) are indicated. See also Figures S3–S7.

To determine whether the excessive neurogenesis is *Pbrm1* cKO microglia‐dependent, we isolated primary microglia and NSPCs from *Pbrm1* cKO and WT mice and established an indirect co‐culture system using Transwell. Immunofluorescence staining of BrdU and Nestin showed significantly increased proliferation of NSPCs co‐cultured with *Pbrm1* cKO microglia (Figure S5E,F), while NSPCs co‐cultured with *Pbrm1* cKO microglia differentiated into a significantly higher number of Tuj1^+^ cells and a lower number of GFAP^+^ cells (Figure S5G,H). To investigate whether PBRM1 deficiency in human microglia affects neurogenesis, we used CRISPR/Cas9‐guided gene editing to delete *PBRM1* in human embryonic stem cells (hESCs), which were then differentiated into human microglia‐like cells (hMGLs) (Figure S6A). Immunofluorescence staining of SOX2 and Ki67 showed a significant increase in the proliferation of organoids co‐cultured with *PBRM1* KO hMGLs (Figure 2E,F and Figure S6B–D), accompanied by a marked elevation in the number of NeuN^+^ cells derived from these co‐cultured organoids (Figure 2E,F). Therefore, these data suggest that loss of microglial PBRM1 promotes the proliferation and neuronal differentiation of NSPCs.

We next investigated whether the function of newborn neurons was altered in *Pbrm1* cKO mice. Retroviral labeling of newborn granule neurons in the DG revealed that *Pbrm1* cKO mice exhibited significantly increased dendritic length and more dendritic branches of RFP‐labeled neurons, suggesting increased dendritic complexity in newborn neurons of *Pbrm1* cKO mice (Figure 2G–I). Whole‐cell patch‐clamp recordings further revealed that the action potential firing frequency of newborn granule neurons showed no significant difference between WT and *Pbrm1* cKO mice, suggesting that the intrinsic excitability of newborn neurons in *Pbrm1* cKO mice was not affected (Figure S7A–D). Synaptic dysfunction has been implicated in the pathogenesis of ASD and anxiety‐related behaviors. We next assessed whether *Pbrm1* deficiency affected synaptic structure and function. Western blotting analysis revealed increased PSD95 expression in *Pbrm1* cKO mice, and Thy1‐GFP reporter mice showed elevated dendritic spine density (Figure S7E–H). Electrophysiological recordings demonstrated an excitatory/inhibitory imbalance characterized by an increased excitatory/inhibitory (E/I) ratio under both 50% and 100% stimulation conditions in cKO mice (Figure S7I,J), suggesting that hippocampal excitatory/inhibitory balance is disrupted in *Pbrm1* cKO mice.

The Wnt signaling pathway, which plays critical roles in NSPCs expansion and differentiation [25], was upregulated in cKO mice (Figure S4D). To investigate whether the Wnt/β‐catenin signaling pathway was affected, we performed Western blotting and found a significant increase in β‐catenin, AKT, and PI3K phosphorylation in the *Pbrm1* cKO hippocampus (Figure 2J). These results suggest activation of Wnt/β‐catenin and PI3K/AKT signaling pathways that may contribute to the enhanced neurogenesis in *Pbrm1* cKO mice.

### 2.4 Inhibition of Neurogenesis Rescues ASD‐Like Behaviors in *Pbrm1* cKO Mice

To determine whether excessive neurogenesis contributes to ASD‐like behaviors in *Pbrm1* cKO mice, we used temozolomide (TMZ), an antimitotic drug that impairs hippocampal neurogenesis (Figure 3A). As expected, TMZ treatment significantly reduced the number of DCX^+^BrdU^+^ newborn neurons and NeuN^+^BrdU^+^ mature neurons in the DG of *Pbrm1* cKO mice to levels comparable to those in WT controls (Figure 3B,C). Mouse body weight was monitored daily, with no significant effect of TMZ on body weight gain observed (Figure 3D). Next, we assessed locomotor activity using the OFT and found that TMZ treatment did not affect general locomotor activity (Figure 3E). Interestingly, TMZ‐treated *Pbrm1* cKO mice spent more time interacting with introduced mice in the social interaction test, suggesting that excessive neurogenesis contributes to the ASD‐like social deficits observed in *Pbrm1* cKO mice (Figure 3F–I).

**FIGURE 3:**
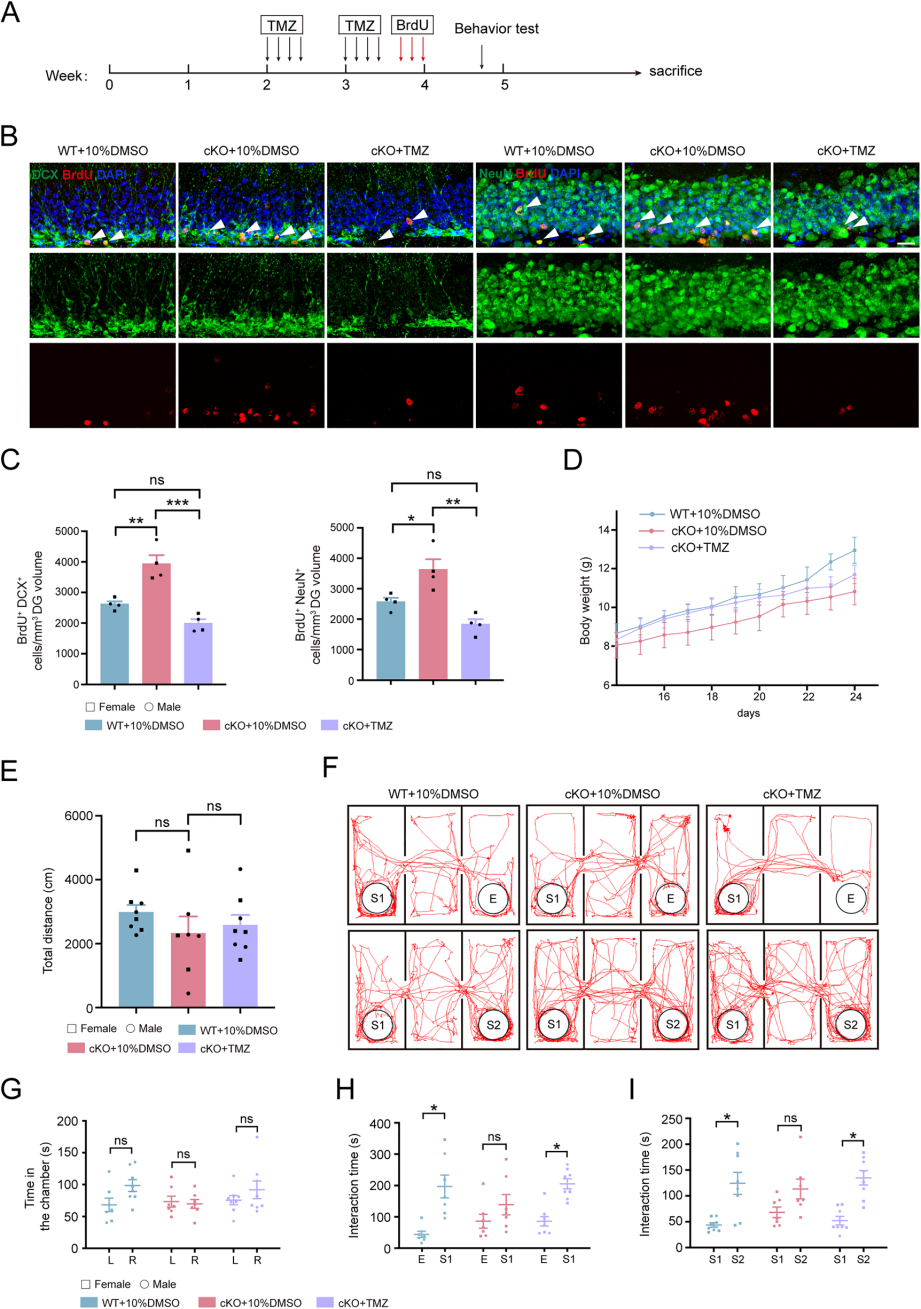
Temozolomide rescues the ASD‐like behaviors in *Pbrm1* cKO mice. (A) Timeline showing the experimental design. (B) Confocal images of DAPI (blue), BrdU (red), and DCX (green) or NeuN (green) immunostaining. Scale bars, 20 µm. (C) The administration of temozolomide decreased the number of DCX^+^BrdU^+^ immature neurons and NeuN^+^BrdU^+^ mature neurons in *Pbrm1* cKO mice. Mean ± SEM. ns, no significant; ^*^ *p*< 0.05; ^**^ *p*< 0.01; ^***^ *p*< 0.001 by one‐way ANOVA followed by Tukey's multiple comparisons test. n = 4 mice per group. Males (circles) and females (squares) are indicated. (D) Body weight of mice was monitored daily during TMZ treatment. TMZ administration did not affect body weight gain. Mean ± SEM. ns, no significant. n = 7–8 mice per group. (E) Quantification of total distance moved in the center zone of WT, *Pbrm1* cKO, and temozolomide‐treated cKO mice in a 10 min test period of the open field test. Temozolomide did not affect the locomotor activity of temozolomide‐treated cKO mice. Mean ± SEM. ns, no significant by Kruskal–Wallis test followed by Dunn's multiple comparisons test. n = 7–8 mice per group. Males (circles) and females (squares) are indicated. (F–I) Representative trajectories (F) and quantifications of time spent in the chambers (G), interaction time from the Stranger 1‐Empty (H) and Stranger 1–Stranger 2 (I) trials in the three‐chamber test. In the three‐chamber test, the application of TMZ ameliorated the impaired sociability and the social novelty in *Pbrm1* cKO mice. Mean ± SEM. ns, no significant, ^*^ *p*< 0.05 by Two‐way ANOVA followed by Šídák's multiple comparisons test. n = 7–8 mice per group. Males (circles) and females (squares) are indicated.

### 2.5 Altered Microglial Clusters in *Pbrm1* cKO Mice

To investigate the cellular and molecular basis underlying microglial PBRM1‐regulated neurogenesis, we conducted a detailed analysis of microglia (5,748 cells) by scRNA‐seq and identified seven microglial clusters (MG0‐MG6) based on their DEG profiles (Figure 4A–C). GO analysis revealed distinct cellular functions of microglial clusters (Figure S8A,B). MG0 represents homeostatic microglia, characterized by high expression of *Tmem119* and *P2ry12* [26, 27]. MG1 is related to chemokine production and cell chemotaxis, marked by *Tnf* and *Nfkbia* [25]. MG2 likely plays a role in synaptic functions and is characterized by high expression of *Rrad* and *Icam1*. MG3 is involved in vasculature development, with specific upregulation of *Cldn5* and *Bsg*. MG4 is responsible for interferon responses, defined by high expression of *Ifit2* and *Ifit3* [28, 29]. MG5 may regulate early developmental processes, characterized by high expression of *Aldoc* and *Clu*. MG6 may be involved in myelination, with high expression of *Tspan2* and *Olig1*.

**FIGURE 4:**
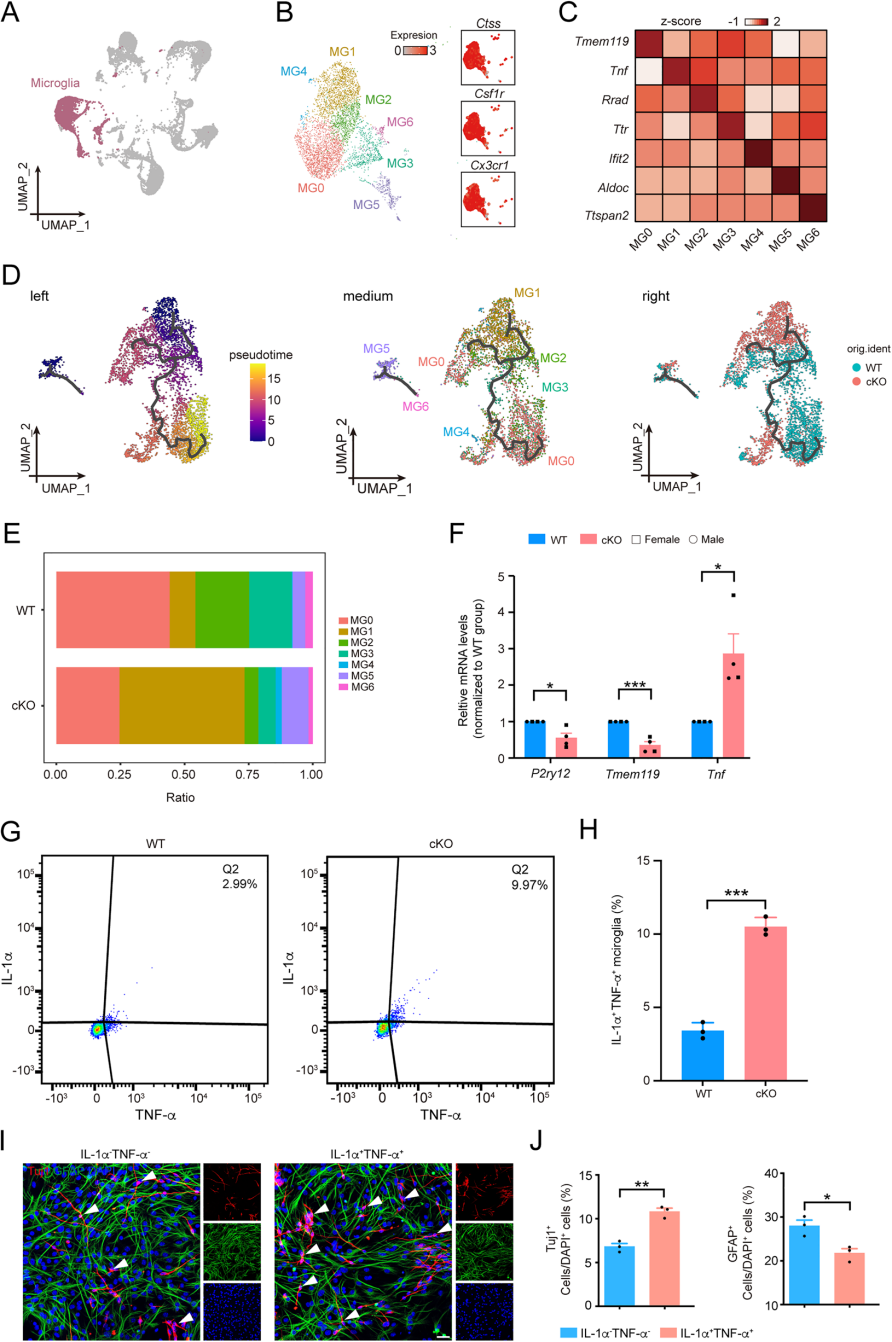
*Pbrm1* cKO microglia disrupts microglial development and homeostasis. (A) Uniform manifold approximation and projection (UMAP) visualization of 22,201 cells, including 10,727 cells from WT hippocampal samples and 11,474 cells from *Pbrm1* cKO hippocampal samples. (B) UMAP visualization of microglia that were divided into 7 subclusters based on the expression of marker genes. (C) Heatmap of normalized expression levels of enriched genes in 7 microglial subclusters. (D) Single‐cell trajectories of microglia by Monocle analysis. Cells are colored by pseudotime trajectories (left panel), clusters (middle panel), or samples (right panel). (E) Bar plot showing relative proportions of each microglial subcluster between WT and cKO samples. (F) Quantitative RT‐PCR analysis showing the relative mRNA levels of *P2ry12, Tmem119*, and *Tnf* in WT and cKO microglia. Compared to WT, cKO microglia displayed significantly lower expression of *P2ry12* and *Tmem119*, but higher expression of *Tnf*. Mean ± SEM. ^*^ *p*< 0.05, ^***^ *p*< 0.001 by Student's *t*‐test or Mann–Whitney test. n = 4 mice per group. Males (circles) and females (squares) are indicated. (G) Representative flow cytometry gating strategy for sorting IL‐1α^+^TNF‐α^+^ microglia from the hippocampus of P15 WT and cKO mice. (H) Quantification of the percentage of IL‐1α^+^TNF‐α^+^ microglia in the hippocampus of P15 WT and cKO mice. Mean ± SEM. ^***^ *p*< 0.001 by Student's *t*‐test. n = 3 mice per group. (I,J) Representative images (I) and quantifications (J) of GFAP (green) and Tuj1 (red) immunostaining of the differentiation of NSPCs that were indirectly co‐cultured with TNF‐α^+^, IL‐1α^+^, TNF‐α^+^IL1‐α^+^ primary microglia in a Transwell system. In contrast to NSPCs that were indirectly co‐cultured with TNF‐α^−^ IL‐1α^−^ cKO microglia, TNF‐α^+^, IL‐1α^+^, TNF‐α^+^IL‐1α^+^ treated NSPCs differentiated into more Tuj1^+^ cells and fewer GFAP^+^ cells. Scale bar, 50 µm. Mean ± SEM. ^*^ *p*< 0.05, ^**^ *p*< 0.01 by Student's *t*‐test. n = 3 independent experiments. See also Figure S8.

We next performed pseudotime analysis to infer the developmental trajectories in each microglial subcluster using the Monocle3 R package. Genes associated with proliferative microglia [26, 29, 30] and early developmental microglia [31], including *Mki67*, *Cenpa*, *Pf4*, *Ube2c*, and *Ms4a7*, exhibited the highest expression levels in the MG1 cluster, which was identified as the developmental starting point (Figure 4D, left and middle panels). We observed that most cKO microglia were enriched at the early stage of the pseudotime trajectory, while most WT microglia were distributed at the middle and terminal stages of the trajectory (Figure 4D, right panel). By calculating relative proportions of each microglial cluster between WT and *Pbrm1* cKO mice, we found that MG1 was significantly increased (48.8%) and MG0 was significantly decreased in *Pbrm1* cKO mice (Figure 4E). Consistently, qRT‐PCR analysis showed decreased expression of MG0‐enriched genes (*P2ry12*, *Tmem119*) and increased expression of the MG1‐enriched gene *Tnf* in CD11b^+^ microglia isolated from *Pbrm1* cKO hippocampus (Figure 4F). Flow cytometry further demonstrated a significant increase in IL‐1α^+^/TNF‐α^+^ double‐positive microglia in *Pbrm1* cKO mice (Figure 4G,H). These data suggest that PBRM1 deficiency results in impairment of microglial development.

Given the above observations of altered microglial clusters in *Pbrm1* cKO mice, we further examined interactions between microglial clusters and NSCs by using the CellChat toolkit. We found that MG1 exhibited the strongest interaction with NSCs (Figure S8C–E), and that CCL, TGF‐β, and galectin signaling pathways might mediate the interaction between the MG1 cluster and NSCs (Figure S8F). These data suggest that the increased neurogenesis following *Pbrm1* loss‐of‐function in microglia may be attributed to the MG1 cluster.

To test whether the MG1 subset drives excessive hippocampal neurogenesis, we isolated IL‐1α^+^ and TNF‐α^+^ microglia using flow cytometry (Figure S8G). When co‐cultured with primary NSPCs in a Transwell system, IL‐1α^+^, TNF‐α^+^, and IL‐1α^+^TNF‐α^+^ microglia significantly promoted neuronal differentiation, evidenced by an increased proportion of Tuj1^+^ cells and a reduced proportion of GFAP^+^ astrocytes (Figure 4I,J). These findings indicate that the MG1‐like microglial subset is sufficient to stimulate NSPCs neuronal lineage commitment, thereby identifying it as the key pathological population mediating the effects of PBRM1 deficiency in microglia.

### 2.6 Microglial CCL5 Drives Excessive Neurogenesis and Social Deficits in *Pbrm1* cKO Mice

To understand the molecular mechanism underlying the interaction between *Pbrm1* cKO microglia and NSPCs, we sorted microglia from the hippocampus of WT and *Pbrm1* cKO mice at P15 using CD11b^+^ magnetic beads and performed bulk RNA sequencing. We identified 221 genes upregulated and 321 genes downregulated in *Pbrm1* cKO microglia compared to those in WT microglia. GO analysis revealed that the upregulated genes were involved in cytokine secretion, chemokine‐mediated signaling, and DNA damage checkpoints, while downregulated genes were enriched in terms related to tissue remodeling (Figure S9A). Next, we performed ATAC‐seq on CD11b^+^ microglia and surprisingly found a significant increase in chromatin accessibility in cKO microglia compared to WT microglia (Figure S10A). Differential ATAC‐seq peaks were located at promoter (20.65%) and intronic/intergenic (42.39%/24.22%) regions (Figure S10B,D), suggesting PBRM1 affects distal regulatory elements, such as enhancers and promoters. Interestingly, GO analysis showed that the upregulated genes were highly enriched for chemokine secretion and chemotaxis regulation (Figure S10C), which were consistent with the bulk RNA‐seq data.

Subsequently, we performed an integrative analysis of gene expression and chromatin accessibility and found 11 overlapping genes among the upregulated ATAC‐seq peaks, the upregulated genes in *Pbrm1* cKO microglia by RNA‐seq, and the upregulated genes in the MG1 cluster by scRNA‐seq (Figure 5A). Among these 11 common genes, we particularly focused on *Ccl5* and *Ccl12*, as they were predicted to mediate microglia‐NSPCs interactions (Figure S8F). Volcano plots (Log_2_fold change ≥ 0.5, *p*< 0.05) from bulk RNA sequencing revealed that the expression of *Ccl5* and *Ccl12* was elevated in *Pbrm1* cKO microglia (Figure 5B). Our scRNA‐seq data showed a significant upregulation of *Ccl5* only in the MG1 cluster in cKO mice (Figure 5C). The expression of *Ccl12* was not significantly increased in the MG1 cluster (Figure 5C). Subsequently, we performed qRT‐PCR and found that *Ccl5* but not *Ccl12* mRNA expression was consistently upregulated in *Pbrm1* cKO microglia (Figure 5D). Also, an ELISA assay confirmed the elevated protein level of CCL5 in *Pbrm1* cKO microglia (Figure 5E).

**FIGURE 5:**
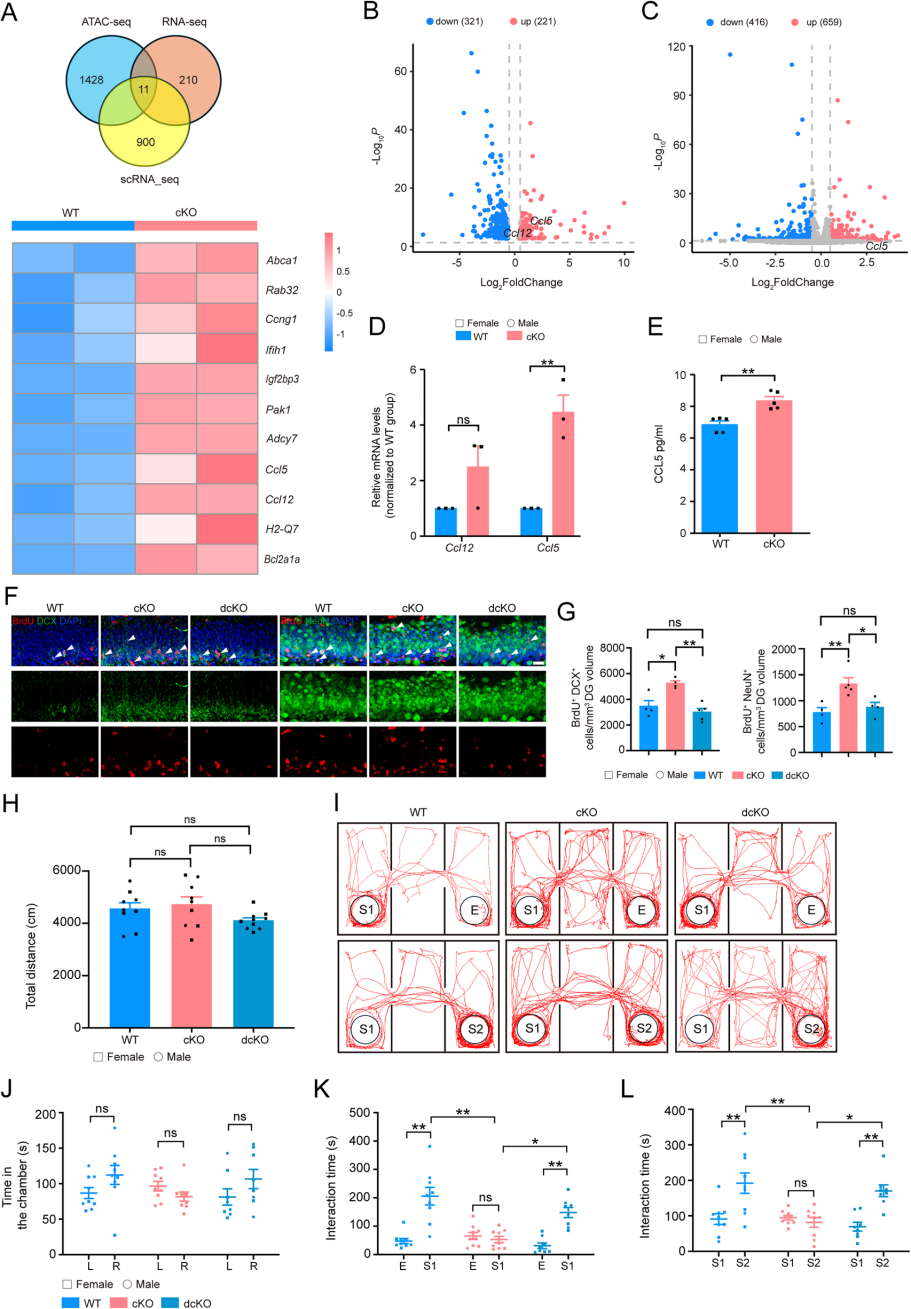
CCL5 is upregulated in *Pbrm1* cKO microglia (A) Venn diagram (upper panel) and heatmaps (lower panel) showing common differentially expressed genes (Log_2_fold‐change ≥ 0.5, *p*< 0.05) in *Pbrm1* cKO microglia identified through scRNA‐seq, ATAC‐seq and RNA‐seq. (B) Volcano plots of differentially expressed genes (RNA‐seq) in cKO microglia at P15 (*n* = 4 experiments analyzed by DESeq2; Log_2_fold‐change ≥ 0.5, *p*< 0.05). (C) Volcano plots of differentially expressed genes (scRNA‐seq) in MG1‐subtype *Pbrm1* cKO microglia at P15 (n = 2 independent experiments analyzed by FindMarkers; Log_2_fold‐change ≥ 0.5, *p*< 0.05). (D) Relative mRNA expression levels of *Ccl5* and *Ccl12* in primary microglia purified from WT and cKO mice at P15 using CD11b^+^ microbeads. Compared to WT, cKO microglia displayed significantly higher expression of *Ccl5*. Mean ± SEM. ns, no significant; ^**^ *p*< 0.01 by Student's *t*‐test. n = 3 mice per group. Males (circles) and females (squares) are indicated. (E) ELISA test showed that CCL5 protein expression was significantly elevated in primary microglia that were isolated from newborn cKO pups. Mean ± SEM. ^**^ *p*< 0.01 by Mann–Whitney test. n = 5 pups per group. Males (circles) and females (squares) are indicated. (F) Representative confocal images of DAPI (blue), BrdU (red), and DCX (green), or NeuN (green) immunostaining of hippocampal tissues from WT, cKO, and dcKO mice. Scale bars, 20 µm. (G) Quantification of DCX^+^BrdU^+^ cells and NeuN^+^BrdU^+^ cells showing that the increased neurogenesis observed in *Pbrm1* cKO mice is significantly restored in dcKO mice. Mean ± SEM. ns, no significant; ^*^ *p*< 0.05; ^**^ *p*< 0.01 by one‐way ANOVA followed by Tukey's multiple comparisons test. n = 4–5 mice per group. Males (circles) and females (squares) are indicated. (H) Quantification of total distance moved in the zone of WT, *Pbrm1* cKO, and dcKO mice in a 10 min test period of the open field test. Mean ± SEM. ns, no significant by one‐way ANOVA followed by Tukey's multiple comparisons test. n = 9–10 mice per group. Males (circles) and females (squares) are indicated. (I–L) Representative trajectories (I) and quantifications of time spent in the chambers (J), interaction time from the Stranger 1‐Empty (K) and Stranger 1–Stranger 2 (L) trials in the three‐chamber test. In the three‐chamber test, genetic deletion of *Ccl5* in *Pbrm1* cKO mice restored impaired sociability and social novelty to levels comparable to those in WT mice. Mean ± SEM. ns, no significant; ^*^ *p*<0.05; ^**^ *p*< 0.01 by Two‐way ANOVA followed by Šídák's multiple comparisons test. n = 9–10 mice per group. Males (circles) and females (squares) are indicated. See also Figures S9–S12.

To determine whether CCL5 can directly act on NSPCs, we examined the expression of its receptors, CCR1 and CCR5, in primary cultured NSPCs. Western blotting analysis showed that CCR1 and CCR5 are expressed in NSPCs, with no significant differences between WT and cKO NSPCs (Figure S11A). We next added recombinant CCL5 to the culture medium of NSPCs for 3 days. We found that exogenous CCL5 significantly increased the numbers of BrdU^+^Nestin^+^ cells and Tuj1^+^ cells but reduced the number of GFAP^+^ astrocytes (Figure S11B,C). To investigate whether the β‐catenin signaling pathway was activated, we performed Western blotting and found that CCL5 significantly increased the expression of β‐catenin and AKT phosphorylation in NSPCs (Figure S11D,E). Meanwhile, we performed stereotaxic injection of recombinant CCL5 protein into the hippocampal DG of P15 mice, with IgG injection serving as the control. We found that CCL5 protein significantly increased the numbers of DCX^+^BrdU^+^ cells and NeuN^+^BrdU^+^ cells in the hippocampus of *Pbrm1* WT mice (Figure S11F,G). Furthermore, blocking CCL5 significantly reduced the proliferation and neuronal differentiation of NSPCs by adding a CCL5 monoclonal antibody in the indirect co‐culture system (Transwell) of *Pbrm1* cKO microglia and NSPCs (Figure S12A,B).

To genetically confirm CCL5 as the critical downstream effector of PBRM1, we generated a microglia‐specific conditional *Pbrm1* and *Ccl5* knockout mouse line by crossing *Pbrm1 ^flox/flox^, Ccl5 ^flox/flox,^* and *Cx3cr1‐Cre* mice to produce *Cx3cr1‐Cre^+/−^*; *Pbrm1^flox/flox;^ Ccl5 ^flox/flox^* mice (*Pbrm1* dcKO). Strikingly, deletion of *Ccl5* completely rescued the hyper‐neurogenic phenotype in *Pbrm1* cKO mice, as demonstrated by a significant reduction in the numbers of BrdU^+^DCX^+^ and BrdU^+^NeuN^+^ cells to levels comparable with WT controls (Figure 5F–G and Figure S12C). We further performed behavioral analyses using *Pbrm1* dcKO mice. We found that genetic deletion of *Ccl5* in *Pbrm1* cKO mice ameliorated social deficits (Figure 5H–L). These results together suggest that microglial CCL5 is a key driver of excessive neurogenesis and social deficits in *Pbrm1* cKO mice.

### 2.7 PBRM1 Binds to *Ccl5* Promoter and Represses its Transcription

The bromodomains of PBRM1 recognize acetylated histones, enabling PBRM1 to regulate gene expression [32, 33]. To investigate whether PBRM1 regulates CCL5 expression in a histone acetylation‐dependent manner, we performed Western blotting to detect histone acetylation levels in primary microglia. Surprisingly, we found that the levels of H3K14ac, H3K27ac, H3K18ac, and H3K56ac were not significantly increased in *Pbrm1* cKO microglia compared to WT (Figure 6A), suggesting that PBRM1's regulation of CCL5 is not associated with global alterations in histone acetylation.

**FIGURE 6:**
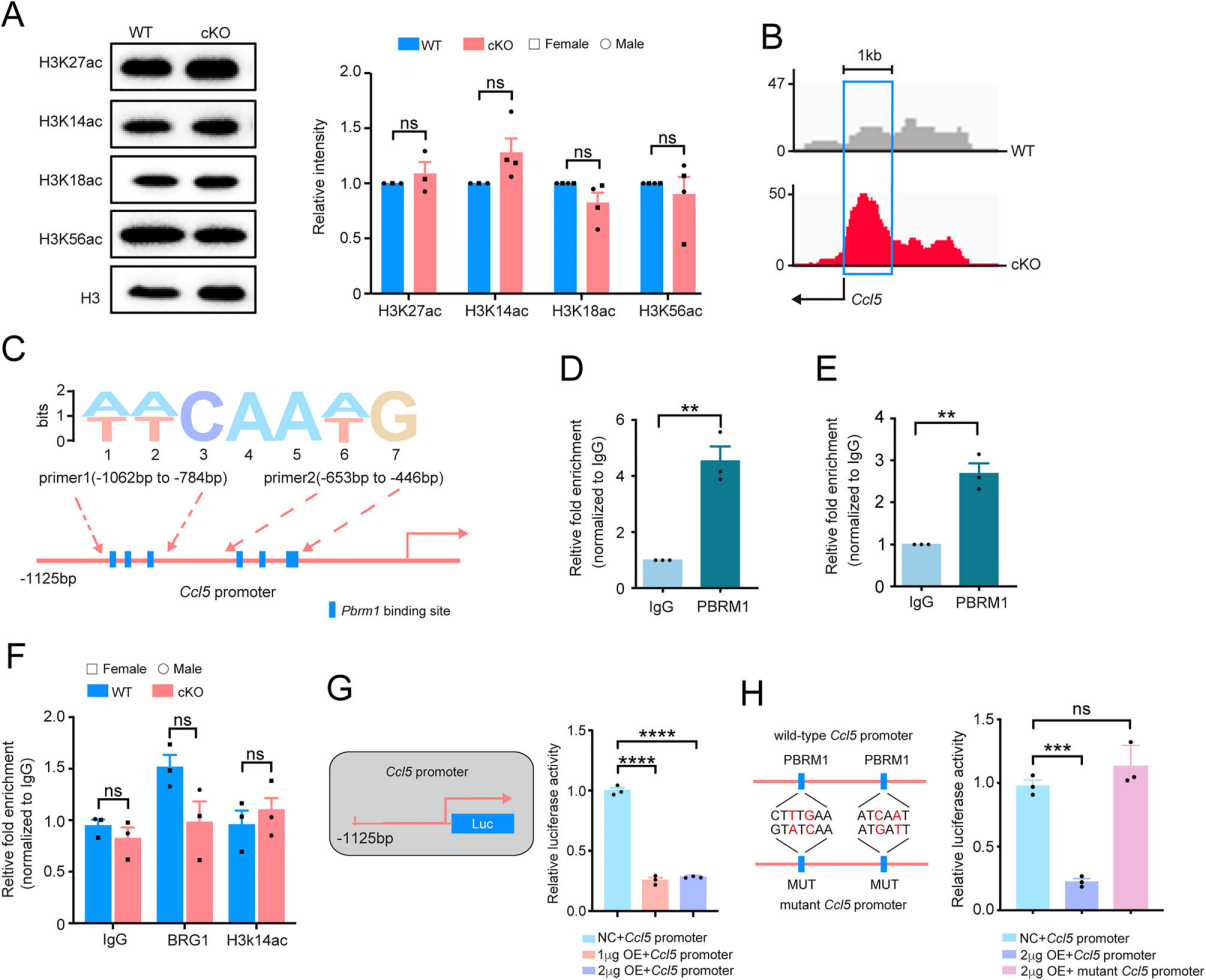
PBRM1 represses the transcription of *Ccl5* and is independent of H3K14ac and BRG1. (A) Western blotting showed that H3K14ac, H3K18ac, H3K56ac, and H3K27ac levels were not altered in *Pbrm*1 cKO microglia. Mean ± SEM. ns, no significant by Student's *t*‐test. n = 3–4 mice per group. Males (circles) and females (squares) are indicated. (B) Genome browser tracks showing ATAC‐seq signals at the *Ccl5* locus in WT and cKO microglia that were purified with CD11b^+^ microbeads from WT and cKO mice at P15. (C) The sequence of the PBRM1 binding motif and ChIP primer pairs for the validation of putative PBRM1 binding sites at the core promoter region of *Ccl5*. ChIP primer pairs 1 and 2 were designed to amplify the [−1062, −784] and [−653, −446] positions relative to the transcription start site of *Ccl5*, respectively. (D‐E) ChIP‐qPCR analysis of PBRM1 binding enrichment in the [−1062, −784] (D) and [−653, −446] (E) positions relative to the transcription start site of *Ccl5*. Mean ± SEM. ^**^ *p*< 0.01 by Student's *t*‐test. n = 3 independent experiments. (F) ChIP‐qPCR for enhancer markers H3K14ac and BRG1 enrichment in the 0‐(‐1) kb upstream region of the *Ccl5* promoter was not significantly increased in cKO microglia. Mean ± SEM. ns, no significant by Student's *t*‐test. n = 3 mice per group. Males (circles) and females (squares) are indicated. (G) Relative luciferase activity in 293T cells that were co‐transfected with the *Ccl5*‐promoter luciferase reporter plasmid and the PBRM1 expression vector. Protein lysates were prepared at 48 h following transfection and then used to measure dual luciferase enzyme activity. Mean ± SEM. ^****^ *p*< 0.0001 by one‐way ANOVA followed by Šídák's multiple comparisons test. n = 3 independent experiments. (H) Relative luciferase activity in 293T cells that were co‐transfected with the PBRM1 expression vector and wild‐type or mutant *Ccl5*‐promoter luciferase reporter plasmid. Protein lysates were prepared at 48 h following transfection and then used to measure dual luciferase enzyme activity. Mean ± SEM. ns, no significant; ^***^ *p*< 0.001 by one‐way ANOVA followed by Šídák's multiple comparisons test. n = 3 independent experiments. See also Figure S10 and S13.

Our earlier ATAC‐seq analysis indeed revealed an unexpected increase in chromatin accessibility within the 0 to −1 kb region of the *Ccl5* promoter in *Pbrm1* cKO microglia (Figure 6B). As PBRM1 has been reported to bind the A(T)A(T)C AAA(T)G DNA sequences (Figure 6C), and multiple putative PBRM1 binding sites were predicted within the −1126 bp region of the *Ccl5* promoter, we then examined whether PBRM1 directly binds to the promoter of *Ccl5*. We performed ChIP‐qPCR assays in the BV2 cell line to analyze the interaction between PBRM1 and its predicted binding sites on the promoter region of *Ccl5* (Figure 6C). As expected, we detected significant enrichment of PBRM1 at two sites within the *Ccl5* promoter (Figure 6D,E), implicating that PBRM1 directly binds to the *Ccl5* promoter.

PBRM1 is a SWI/SNF complex subunit, and BRG1 is another core subunit of the SWI/SNF complex responsible for ATP hydrolysis and energy provision [34, 35]. We next examined whether this binding involves other SWI/SNF components or local histone acetylation. ChIP‐qPCR assays showed no significant enrichment of BRG1 at the *Ccl5* promoter in *Pbrm1* cKO microglia (Figure 6F). These results indicate that PBRM1 may regulate *Ccl5* transcription through a mechanism independent of the canonical manner. We further assessed H3K14ac enrichment at the *Ccl5* promoter. A trend toward increased H3K14ac was observed in *Pbrm1* cKO microglia, though without statistical significance (Figure 6F). Public ChIP‐seq datasets showed variable H3K14ac occupancy at the *Ccl5* promoter across different contexts (Figure S13). Collectively, these findings suggest that PBRM1 primarily represses *Ccl5* transcription in microglia through a mechanism largely independent of BRG1 and H3K14ac.

To determine whether *Ccl5* is a downstream target of PBRM1, we cloned a 1126 bp fragment of the *Ccl5* promoter containing PBRM1 binding sites into a dual‐luciferase reporter vector (CD511), which was used to assess CCL5 transcriptional activity by measuring luciferase activities. We found that overexpression of PBRM1 significantly inhibited the expression of Renilla luciferase (R‐Luc) via the *Ccl5* promoter (Figure 6G). To assess whether PBRM1 binds to the *Ccl5* promoter through the A(T)A(T)C AAA(T)G sequences, we mutated the A(T)A(T)C AAA(T)G sequences within the *Ccl5* promoter of the R‐Luc reporter and found that the mutated *Ccl5*‐promoter construct abolished the inhibited R‐Luc activity induced by PBRM1 overexpression (Figure 6H). These findings suggest that PBRM1 represses *Ccl5* transcription through binding to the *Ccl5* promoter via the A(T)A(T)C AAA(T)G sequence.

### 2.8 CCL5‐CCR1/5 Activates β‐catenin Signaling to Drive Excessive Neurogenesis in *Pbrm1* cKO Mice

CCR1 and CCR5 are the primary receptors for CCL5 and are involved in regulating immune and inflammatory responses [36, 37]. To investigate whether CCL5 binds to CCR1 and CCR5 to drive excessive neurogenesis, we added FDA‐approved CCR1 and CCR5 inhibitors (BX471 and maraviroc, respectively) [38, 39] into a Transwell system in which microglia and NSPCs were indirectly co‐cultured. Inhibition of either CCR1 or CCR5 significantly reduced the proliferation of NSPCs co‐cultured with *Pbrm1* cKO microglia, as evidenced by a decrease in the number of Nestin^+^BrdU^+^ cells (Figure 7A,C). Furthermore, CCR1 and CCR5 inhibitors also significantly decreased the number of Tuj1^+^ neurons and increased the number of GFAP^+^ glial cells in the group of NSPCs co‐cultured with *Pbrm1* cKO microglia (Figure 7B,D). Next, we tested whether the CCR1/5 inhibitors decreased the expression of β‐catenin, phosphorylated β‐catenin, and phosphorylated AKT in NSPCs. Western blotting analysis revealed that individual inhibition of CCR1 or CCR5 failed to reduce the elevated levels of phosphorylated β‐catenin and phosphorylated AKT pathways in NSPCs co‐cultured with *Pbrm1* cKO microglia (Figure S14A,B). However, simultaneous inhibition of both receptors significantly reduced the levels of phosphorylated β‐catenin (Y552), phosphorylated AKT, and phosphorylated PI3K (Figure 7E). These results indicate that CCL5 activates β‐catenin signaling through the CCR1/5‐PI3K/AKT pathway.

**FIGURE 7:**
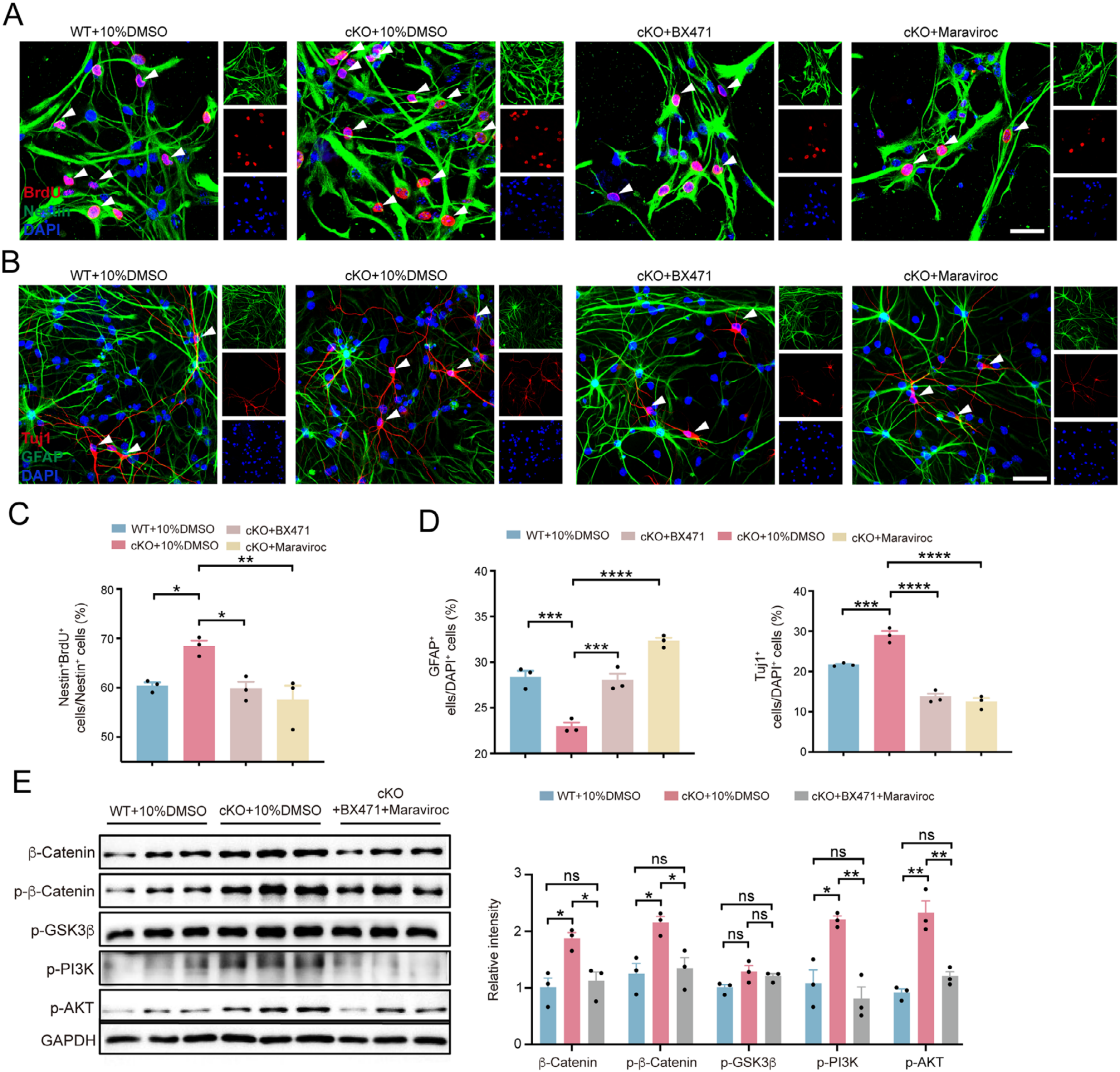
CCR1/5 antagonist inhibits excessive neurogenesis through the β‐catenin signaling pathway. (A) Representative images of DAPI (blue), Nestin (green), and BrdU (red) immunostaining. Scale bars: 20 µm. (B) Representative images of DAPI (blue), GFAP (green), and Tuj1 (red) immunostaining. Scale bars, 20 µm. (C) Quantification analysis of the ratio of the number of Nestin^+^BrdU^+^ NSPCs that were indirectly co‐cultured with *Pbrm1* cKO microglia in a Transwell system. The number of Nestin^+^BrdU^+^ NSPCs was decreased in the CCR1/5 antagonist group but not in the DMSO‐treated group. Mean ± SEM, ^*^ *p*< 0.05, ^**^ *p*< 0.01 by one‐way ANOVA followed by Šídák's multiple comparisons test. n = 3 independent experiments. (D) Quantification analysis of the ratio of the number of GFAP^+^ astrocytes and Tuj1^+^ neurons. In contrast to DMSO‐treated NSPCs that were indirectly co‐cultured with *Pbrm1* cKO microglia, maraviroc‐ or BX471‐ treated NSPCs differentiated into fewer Tuj1^+^ cells and more GFAP^+^ cells. Mean ± SEM. ^***^ *p*< 0.001, ^****^ *p*< 0.0001 by one‐way ANOVA followed by Šídák's multiple comparisons test. n = 3 independent experiments. (E) Western blotting showed the expression levels of β‐catenin, p‐β‐catenin, p‐GSK3β, p‐AKT, and p‐PI3K in NSPCs that were indirectly co‐cultured with *Pbrm1* cKO microglia in a Transwell system, treated with BX471 and maraviroc. Mean ± SEM. ns, no significant; ^*^ *p*< 0.05; ^**^ *p*< 0.01 by one‐way ANOVA followed by Tukey's multiple comparisons test or Kruskal–Wallis test followed by Dunn's multiple comparisons test. n = 3 independent experiments. See also Figure S14.

To validate these findings in vivo, we intraperitoneally injected maraviroc and BX471 into *Pbrm1* cKO mice and assessed neurogenesis by BrdU labeling and immunostaining. We found that pharmacological inhibition of CCR1/5 significantly reduced the excessive numbers of Nestin‐GFP^+^BrdU^+^ cells, DCX^+^BrdU^+^ cells, and NeuN^+^BrdU^+^ cells in the hippocampus of *Pbrm1* cKO mice (Figure S14C,D). Collectively, these in vitro and in vivo findings support that the CCL5/CCR1/5 axis drives excessive neurogenesis in *Pbrm1* cKO mice.

### 2.9 Pharmacological Inhibition of CCR1/5 Rescues Autism‐Like Behaviors in *Pbrm1* cKO Mice

To test whether pharmacological inhibition of CCR1/5 could ameliorate the anxiety and autism‐like behaviors in *Pbrm1* cKO mice, we applied maraviroc and BX471 daily until the start of behavioral assays. Treatment with maraviroc and BX471 significantly increased the time and entries in the center of the open field (Figure 8A,B). EPM tests showed that administration of maraviroc and BX471 significantly increased the time in the open arms (Figure 8C,D), further suggesting that maraviroc and BX471 rescued the anxiety‐like behavior associated with the loss of *Pbrm1*. *Pbrm1* cKO mice under the administration of maraviroc and BX471 spent significantly more time interacting with introduced mice in the social interaction test, suggesting pharmacological inhibition of CCR1/5 improves the sociability of *Pbrm1* cKO mice (Figure 8E–H). Treatment with maraviroc and BX471 significantly decreased the number of marbles buried in *Pbrm1* cKO mice (Figure 8I).

**FIGURE 8:**
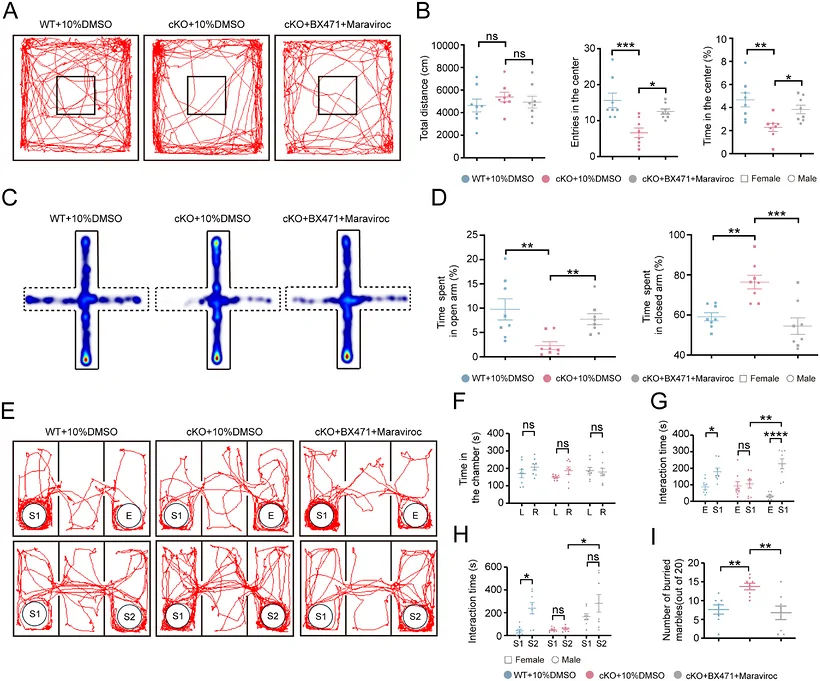
Maraviroc and BX471 alleviate autism‐like behaviors in *Pbrm1* cKO mice (A,B). Representative trajectories (A) and quantification (B) of total distance moved, time spent, and entries in the center zone of *Pbrm1* WT, cKO, and maraviroc‐treated, BX471‐treated cKO mice in a 10 min test period of the open field test. The black line and square represent the center zone. Maraviroc and BX471 significantly increased the time and entries in the center zone of cKO mice. Mean ± SEM. ns, no significant; ^*^ *p*< 0.05; ^**^ *p*< 0.01; ^***^ *p*< 0.001 by one‐way ANOVA followed by Šídák's multiple comparisons test. n = 8 mice per group. Males (circles) and females (squares) are indicated. (C,D) Representative heat maps (C) and quantification (D) of time spent in open arms and closed arms in the elevated plus maze test. The time in the open arms was significantly increased, and the time in the closed arms was significantly decreased in the *Pbrm1* cKO mice treated with maraviroc and BX471. Mean ± SEM. ^**^ *p*< 0.01, ^***^ *p*< 0.001 by one‐way ANOVA followed by Šídák's multiple comparisons test or Kruskal–Wallis test followed by Dunn's multiple comparisons test. n = 8 mice per group. Males (circles) and females (squares) are indicated. (E‐H) Representative trajectories (E) and quantifications of time spent in the chambers (F), interaction time from the Stranger 1‐Empty (G) and Stranger 1–Stranger 2 (H) trials in the three‐chamber test. In the three‐chamber test, the application of maraviroc and BX471 ameliorated the impaired sociability but not the social novelty in *Pbrm1* cKO mice. Mean ± SEM. ns, no significant; ^*^ *p*< 0.05; ^**^ *p*< 0.01; ^****^ *p*< 0.0001 by two‐way ANOVA followed by Šídák's multiple comparisons test. n = 8 mice per group. Males (circles) and females (squares) are indicated. (I) Quantification of the marble burying score per group. Data showed that the number of buried marbles decreased in *Pbrm1* cKO mice treated with maraviroc and BX471. Mean ± SEM. ^**^ *p*< 0.01 by one‐way ANOVA followed by Šídák's multiple comparisons test. n = 8 mice per group. Males (circles) and females (squares) are indicated.

## 3 Discussion

Although a combination of genetic and environmental risk factors is widely recognized to contribute to ASD phenotypes, the molecular mechanisms that connect disease‐associated variants with abnormal neurodevelopment remain incompletely understood [40, 41]. Rare missense variants in *PBRM1* have been implicated in ASD risk [9]. This study identifies a previously unrecognized role of microglial PBRM1 in brain homeostasis, neurogenesis, and behaviors. Microglial PBRM1 deficiency drives a shift from homeostatic microglia toward a TNF‐α^+^/IL‐1α^+^ subcluster, resulting in elevated CCL5 secretion. Increased CCL5 subsequently activates CCR1/5‐dependent PI3K/AKT/β‐catenin signaling in NSPCs, thereby enhancing their proliferation and neuronal differentiation. This excessive neurogenesis was accompanied by ASD‐like social deficits and repetitive behaviors in *Pbrm1* cKO mice. Mechanistically, we further demonstrate that PBRM1 directly binds to the *Ccl5* promoter and represses its transcription independently of the canonical PBAF/SWI/SNF complex. Together, these findings reveal that PBRM1 is essential for maintaining microglial homeostasis during brain development and suggest that disruption of microglial‐derived CCL5 contributes to aberrant neurogenesis and subsequent ASD‐like behavioral abnormalities.

Accumulating genetic evidence has linked *PBRM1* to multiple neurodevelopmental and psychiatric disorders, including ASD and major depressive disorder. Consistent with this association, human genetic analyses indicate that *PBRM1* is highly constrained for loss‐of‐function mutations (RVIS = −2.287, 2.23rd percentile) [42], supporting an essential physiological role. In agreement with these observations, microglia‐specific PBRM1 deletion in mice leads to ASD‐like and anxiety‐like behaviors. Loss‐of‐function (LOF) mutations in PBRM1 frequently occur in the bromodomains, thereby impairing chromatin remodeling activity in clear‐cell renal cell carcinoma [32, 43]. Similar structural perturbations may also compromise PBRM1 function during neurodevelopment. Notably, previous studies in macrophages have further demonstrated that PBRM1‐containing PBAF complexes regulate inflammatory gene programs through transcription factors such as NF‐κB [34]. Extending these findings to the central nervous system, our single‐cell transcriptomic analyses revealed that deletion of *Pbrm1* leads to altered microglial clusters, as evidenced by an increased percentage of MG1 but a reduced percentage of MG0. Dysregulated genes in MG1 are enriched in pathways related to cell chemotaxis, immune response, and chemokine production, highlighting the important role of microglial PBRM1 in maintaining brain homeostasis during development.

Microglia are increasingly recognized for their essential role in modulating NSPCs within neurogenic niches, particularly by regulating proliferation and differentiation [44, 45, 46]. Our findings indicate that TNF‐α^+^IL‐1α^+^ microglia induced by *Pbrm1* deficiency promote proliferation and neuronal differentiation of NSPCs. Moreover, inhibiting excessive neurogenesis in *Pbrm1* cKO ameliorates ASD‐like behavioral deficits, supporting a link between aberrant neurogenesis and behavioral dysfunction. Although no significant sex‐dependent effects were detected in social behavior or neurogenesis, sex‐dependent differences cannot be completely excluded. Autism is characterized by early brain overgrowth, and male children with ASD (aged 2–16 years) have been reported to have 67% more neurons in the prefrontal cortex than control children, indicating a potential link between excess neurogenesis and increased brain volume in ASD [47]. In contrast, *Shank3*‐deficient mice exhibit decreased overall neurogenesis due to the dormancy of quiescent NSPCs in the SVZ and hippocampal subgranular zone [48]. Altered neurogenesis may lead to abnormal brain growth and an imbalance in excitatory and inhibitory synapses, which are associated with social and behavioral deficits observed in ASD [49, 50, 51]. Beyond ASD‐like phenotypes, *Pbrm1* cKO mice also exhibited anxiety‐like behaviors, highlighting a potential overlap between anxiety‐like and ASD‐related behaviors, consistent with the substantial clinical overlap between ASD and anxiety disorders, which share abnormalities in synaptic function and excitatory–inhibitory balance [52, 53, 54]. Supporting this interpretation, we observed increased dendritic spine density, elevated excitatory/inhibitory synaptic ratios, and hyperactivation of the PI3K/AKT/β‐catenin signaling pathway in *Pbrm1* cKO mice. Moreover, recent studies have identified PBRM1 within a shared interaction module containing high‐confidence ASD genes such as CHD8 and CTNNB [9]. Nevertheless, the cellular and molecular mechanisms underlying how excessive hippocampal neurogenesis contributes to ASD‐like behaviors in microglial *Pbrm1*‐deficiency mice warrant further investigation.

PBRM1 is an essential component of the SWI/SNF chromatin remodeling complex, which plays a crucial role in regulating gene expression by modulating chromatin accessibility. In cancer, PBRM1 inactivation frequently disrupts SWI/SNF nucleosome remodeling, leading to alterations in the chromatin landscape, including changes in histone acetylation at promoters and enhancers [32, 55, 56, 57]. Consistent with its established chromatin‐remodeling function, our ATAC‐seq analysis revealed a significant increase in chromatin accessibility in *Pbrm1* cKO microglia, particularly within promoter and intronic/intergenic regions. These findings indicate that PBRM1 broadly constrains the chromatin landscape of developing microglia. Unexpectedly, global histone acetylation remained largely unchanged despite extensive chromatin remodeling. Together with the heterogeneous H3K14ac occupancy observed in publicly available ChIP‐seq datasets, these observations suggest that recruitment of PBRM1 to chromatin is not solely determined by histone acetylation status and is likely influenced by additional regulatory mechanisms.

Previous studies have shown that loss of PBRM1 leads to the formation of ectopic complexes containing ARID2 and BRG1, which aberrantly activate potent transcription factors such as NF‐κB [57]. Surprisingly, neither our ChIP‐qPCR experiments nor analyses of publicly available BRG1 ChIP‐seq datasets detected BRG1 enrichment at the *Ccl5* promoter, indicating that transcriptional regulation of *Ccl5* by PBRM1 is largely independent of the canonical PBAF/SWI/SNF complex. We cannot exclude the possibility that residual BRG1/ARID2 complexes contribute indirectly to *Ccl5* activation through enhancer remodeling or by facilitating recruitment of inflammatory transcription factors such as NF‐κB. Nevertheless, our combined ATAC‐seq, ChIP‐qPCR, and luciferase assays demonstrate that PBRM1 directly binds the *Ccl5* promoter via the A(T)A(T)C AAA(T)G motif and represses its transcription independently of BRG1 and H3K14ac. Although no global changes in histone acetylation were detected, the modest increases in H3K14ac at the *Ccl5* promoter may reflect recruitment of acetylated transcription factors (NF‐κB, STAT, or IRF family members), together with their associated coactivators [58, 59, 60, 61]. Given that PBRM1 bromodomains can recognize acetylated non‐histone proteins such as p53 [32, 62], these changes may reflect PBRM1‐mediated recruitment of acetylated transcription regulators. Collectively, our findings support a model in which PBRM1 represses Ccl5 transcription primarily through sequence‐specific promoter binding, whereas bromodomain‐mediated recognition of acetylated proteins serves to reinforce or fine‐tune this regulatory process. Nevertheless, further studies are required to define the role of PBRM1 as a transcriptional regulator in microglia under both physiological and pathological conditions.

Clinical studies have consistently reported elevated plasma levels of CCL5 in children with ASD and attention‐deficit/hyperactivity disorder, suggesting a potential link between CCL5 and neurodevelopmental conditions [63, 64]. Notably, CCL5 contributes to recruitment of immune cells into the brain, and its elevated levels are known to exacerbate neuroinflammation, potentially increasing ASD risk [65, 66, 67]. Beyond its well‐recognized immunomodulatory functions, our study identifies a previously unappreciated role for CCL5 in directly regulating neural stem/progenitor cell behavior. Specifically, CCL5 activated CCR1 and CCR5 receptors on NSPCs, leading to stimulation of the PI3K/AKT/β‐catenin signaling cascade and promoting both cellular proliferation and neuronal differentiation. These observations are consistent with recent reports demonstrating neurotrophic properties of CCL5, including enhancement of hippocampal dendritic spine formation and cellular energy metabolism through PI3K/AKT signaling [68, 69]. Given that synaptic dysfunction, particularly abnormalities in synaptic plasticity, spine density, and energy metabolism, are increasingly recognized as a key pathological feature of ASD [70, 71, 72, 73], further studies are required to determine whether and how elevated CCL5 triggers synaptic deficits in *Pbrm1*‐deficient mice.

Despite substantial advances in understanding ASD genetics, effective mechanism‐based pharmacological therapies remain limited. Notably, CCR1 and CCR5 antagonists, including BX471 and maraviroc, have already demonstrated favorable safety profiles in other clinical settings [38, 74, 75]. They represent attractive candidates for therapeutic repurposing. In this study, pharmacological inhibition of CCR1/5 attenuated excessive hippocampal neurogenesis and ameliorated ASD‐like behaviors in *Pbrm1* cKO mice. However, the treatment failed to completely restore either neurogenic or behavioral phenotypes, indicating that dysregulation of the CCL5–CCR1/5 pathway alone is insufficient to account for the full spectrum of pathological changes induced by PBRM1 deficiency. Consistent with this interpretation, our scRNA‐seq analysis revealed enrichment of the TGF‐β and galectin signaling pathways in *Pbrm1* cKO microglia, both of which have established roles in regulating NSPCs maintenance, neuroblast migration, and neuronal differentiation within the neurogenic niche [76, 77, 78, 79]. These findings support a model in which microglial PBRM1 deficiency perturbs multiple intercellular signaling networks that collectively drive abnormal neurogenesis and behavioral dysfunction, highlighting both the biological complexity of ASD and the need for multi‐target therapeutic strategies.

## 4 Conclusion

Our study identifies microglial PBRM1 as a critical regulator of NSPC proliferation and neuronal differentiation through modulation of microglia–NSPC communication. By establishing the PBRM1–CCL5–CCR1/5 signaling axis as a critical pathway linking microglial dysfunction to excessive neurogenesis and ASD‐like behaviors, these findings provide mechanistic insight into the contribution of chromatin‐remodeling defects to neurodevelopmental disorders. More broadly, they highlight the CCL5–CCR1/5 pathway as a potential therapeutic target for ASD associated with PBRM1 dysfunction.

## 5 Experimental Sections

### 5.1 Animals

All procedures involving animals were performed under the ethical guidelines and received approval from the Animal Committee of the Institute of Zoology, Chinese Academy of Sciences (IOZ‐IACUC‐2024‐176). Microglia‐specific *Pbrm1* conditional mice (*Pbrm1* cKO) were generated by crossing *Pbrm1 ^flox/fl^* ^ox^ mice (Jax strain: 029049) with the *Cx3cr1‐Cre* (Jax strain: 025524) line. The *Ccl5 ^flox/fl^* ^ox^ mice (S‐CKO‐04958, Saiye (Suzhou) Biotechnology Co., Ltd, China) were intercrossed with *Pbrm* cKO mice to generate microglia‐specific *Pbrm1* and *Ccl5* double conditional knockout (dcKO) mice. The Nestin‐GFP transgenic mouse line (Jax strain: 033927) used in this study was originally generated by Enikolopov and colleagues [80]. This mouse line was generously provided by the laboratory of Prof. Weixiang Guo at the Institute of Genetics and Developmental Biology, Chinese Academy of Sciences. The Thy1‐GFP mouse line was generously provided by Fengquan Zhou's laboratory at Johns Hopkins School of Medicine. Both male and female mice were included in this study. All animals were housed in a specific‐pathogen‐free facility under a 12 h light/dark cycle.

### 5.2 Cell Lines

The hESCs (WA09) were generously provided by Prof. Baoyang Hu (Institute of Zoology, Chinese Academy of Sciences) and cultured on Matrigel (356230, BD Biosciences, USA)‐coated plates in mTeSR E8 medium (05990, STEMCELL Technologies, Canada). BV2 microglia cell line (CL‐0493) and human embryonic kidney 293 cells (HEK 293T) were cultured in complete medium (DMEM supplemented with 10% fetal bovine serum and 1% penicillin‐streptomycin).

### 5.3 *PBRM1* Knock‐Out in hESCs

Two sgRNAs targeting PBRM1 were designed using the online CRISPR tool at http://crispr.mit.edu [81]. Approximately 1 × 10^6^ single hESCs were electroporated with 7 µg of each sgRNA plasmid using a Lonza Nucleofector II device (program CM115). After 36 h post‐transfection, GFP^+^ cells were sorted and seeded as single cells. The expanded clones were subsequently screened by PCR for mutations at the PBRM1 locus.

### 5.4 Brain Organoid Culture

Brain organoids were established using the STEMdiff Cerebral Organoid Kit (08570, STEMCELL Technologies, Canada) according to the manufacturer's protocol. In brief, hESCs were dissociated into single cells with 0.5× TrypLE (12604021, Gibco, USA) and seeded in a 96‐well plate (9 000 cells per well) to allow embryoid body (EB) formation [82]. The culture medium was changed every 4 days, and the organoids were harvested on day 42 for subsequent analysis

### 5.5 Cell Isolation and Culture

#### 5.5.1 Primary Microglia

Primary microglia were dissected from the hippocampus of postnatal (day 1–5) mouse pups. The cell suspension was cultured in complete medium for 2 weeks with medium changes every 5 days. Microglia were harvested by shaking the flasks at 170 rpm for 1–4 h at 37°C [83]. The cells were collected from the supernatant and used for subsequent assays.

#### 5.5.2 Differentiation of Human MGLs

For EB formation, hESCs were dissociated, counted, and seeded at 3 × 10^4^ cells/100 µL in U‐bottom 96‐well plates with ROCK inhibitor Y27632. EBs were transferred to 6‐well plates after 24 h and sequentially cultured in S1–S4 media containing combinations of FGF2, BMP4, Activin A, VEGF, LiCl, G‐CSF, TPO, FLT3L, SCF, IL‐3, and IL‐6 under low‐oxygen or normoxic conditions to generate hematopoietic progenitor cells (HPCs). For MGL differentiation, CD43^+^ HPCs were flow‐sorted and seeded onto PDL/Matrigel‐coated 6‐well plates in M1 medium (HPC basal medium plus M‐CSF, IL‐34, TGF‐β1, G‐CSF, IL‐3, GM‐CSF, FLT3L). After 6 days, cells were cultured in M2 medium (HPC basal medium plus M‐CSF, IL‐34, TGF‐β1). On day 12, cells were transferred to M3 medium with additional CD200 and CX3CL1. Medium was refreshed every two days, and cells matured by day 18, yielding functional human microglia‐like cells for downstream applications.

#### 5.5.3 Primary NSPCs

Primary NSPCs were isolated from the hippocampus of postnatal (day 0–2) WT pups and cultured according to previously reported protocols [84, 85]. In brief, the dissected hippocampal tissues were digested with TrypLE. The NSPCs were cultured in neurobasal medium (10888022, Gibco, USA) supplemented with 20 ng/mL epidermal growth factor (EGF), 2% B27(17504044, Gibco, USA), 20 ng/mL basic fibroblast growth factor (bFGF), 1% N2 (17502048, Gibco, USA), and 2 mM GlutaMAX (35050061, Gibco, USA).

For proliferation analysis, cells were labeled with 20 µM BrdU (B5002, Sigma–Aldrich, USA) for 6–8 h. Following fixation, immunostaining was performed. For differentiation, cells were grown in proliferation medium for 24 h, followed by 3–4 days of culture in differentiation medium. For the co‐culture system, NSPCs were isolated from the hippocampus and cultured in the bottom of a Costar Transwell 24‐well plate (3470, Costar Corning, USA) with differentiation medium. Microglia were generated from the hippocampus and grown on the upper chambers of the same Costar Transwell 24‐well plate. For CCL5 stimulation experiments, NSPCs were pre‐treated for 12 hours with EGF/bFGF‐free medium and treated with 100 ng/mL CCL5 (250‐070‐20, Proteintech, USA) for 30 min before subsequent analyses.

### 5.6 ELISA

Supernatants from primary microglial cultures were collected and assayed for CCL5 using a commercial ELISA kit (Abebio, AE51709MO, China). All samples were tested in triplicate according to the manufacturer's protocol.

### 5.7 Quantitative Real‐Time PCR

Total RNA was isolated from cells or tissues. 1 µg of total RNA was reverse transcribed into cDNA using a reverse transcriptase kit (AT3111, TransGen Biotech, China). Real‐time PCR was performed using Hieff qPCR SYBR Green Master Mix (11201ES08, Yeasen, China). Gene expression levels were normalized to *Gapdh*. All primers are listed in Table S1.

### 5.8 Immunofluorescence Staining

Immunofluorescence staining was carried out following a previously established protocol [83]. For staining, 40 µm free‐floating sections were blocked with 5% donkey serum (SL050, Solarbio, China) and incubated with primary antibodies overnight at 4°C. After washing, sections were incubated with Alexa Fluor‐conjugated secondary antibodies for 2 h at room temperature.

### 5.9 Drug Treatment in the Mice

The CCR5 inhibitor (maraviroc) and the CCR1 inhibitor (BX471) were purchased from Lebo. Mice aged 8–10 weeks received maraviroc (50 mg/kg) and BX471 (20 mg/kg) or vehicle (95% oil and 5% DMSO) by intraperitoneal injection every day until the start of behavioral analysis. For experiments involving inhibition of adult neurogenesis, temozolomide (HY‐17364, MCE, China) was administered at 25 mg/kg via subcutaneous injection. Injections were administered twice daily on postnatal days 14–17 and 21–24 [80, 86].

### 5.10 BrdU Incorporation Assay

To assess NSPC proliferation in the SVZ and DG, P15 mice received a single BrdU injection (100 mg/kg, i.p.) and were sacrificed 2 h later. For differentiation analysis in the olfactory bulb and DG, BrdU (50 mg/kg, i.p.) was given twice daily from P9 to P11, and brains were collected 4 days after the last injection to analyze early neuronal differentiation of proliferating cells. BrdU was detected following HCl‐mediated denaturation and immunostaining using the protocol described above.

### 5.11 Retrovirus Production

RFP‐expressing Retrovirus was produced following a previously described protocol [87]. In brief, HEK 293T cells were transfected with the retroviral vector using the calcium phosphate method. The medium was harvested daily for 5 consecutive days, starting 24 h after transfection. The virus was concentrated by ultracentrifugation (72 000 × *g*, 4°C) and resuspended in PBS for subsequent experiments.

### 5.12 Stereotaxic Injection

In vivo retroviral grafting was performed following a previously reported protocol [87]. In brief, 1.5 µL of 1 × 10^8^ genomic particles (gp)/ µL retrovirus was stereotaxically delivered into the DG using the following coordinates relative to bregma: anteroposterior (AP), −2 mm; mediolateral (ML), ±1.7 mm; dorsoventral (DV), −1.9 mm from the dura. In addition, 2 µL of recombinant protein CCL5 (20 ng/µL) was unilaterally administered into the DG, with the contralateral DG receiving an equivalent volume of IgG solution serving as an internal control. For CCL5 injection, the stereotaxic coordinates were AP, −1.7 mm; ML, ±1.2 mm; DV, −1.5 mm from the dura. Dendritic complexity was quantified by Sholl analysis as described previously [88].

### 5.13 Electrophysiology

4‐ to 6‐week‐old mice were anesthetized with isoflurane. Brain sections were quickly immersed in ice‐cold, oxygenated (95% O_2_/5% CO_2_) sucrose‐based cutting solution containing (in mM): 110 sucrose, 2.5 KCl, 25 NaHCO_3_, 1.3 NaH_2_PO_4_, 7 MgCl_2_, 0.5 CaCl_2_, and 25 glucose (pH 7.4; 295 mOsm). Slicing was performed using a vibratome (Leica VTS 1200S) in ice‐cold slicing solution. Slices were then equilibrated in oxygenated artificial cerebrospinal fluid (ACSF; in mM: 125 NaCl, 2.5 KCl, 25 NaHCO_3_, 1.3 NaH_2_PO_4_, 1.3 MgCl_2_, 2.5 CaCl_2_, and 25 glucose; pH 7.4) for at least 1 h at 30°C prior to recording.

Neurons in the DG were visualized using IR‐DIC optics on an inverted Olympus BX51WI microscope. Whole‐cell recordings were obtained using a Multiclamp 700B amplifier and Digidata Digitizer 1550B (Molecular Devices Corporation, Sunnyvale, CA, USA). The signals were sampled at 10 kHz and filtered at 2 kHz. The intrinsic electrophysiological properties of DG neurons were assessed in current‐clamp mode in the presence of CNQX (10 µM), AP5 (50 µM), and Bicuculline (10 µM). The pipette solution contained (in mM): 130 K‐gluconate, 10 HEPES, 5 KCl, 4 MgCl_2_, 0.2 EGTA, 4 MgATP, 0.3 NaGTP, 10 Na_2_‐Phosphocreatine. Resting membrane potential (RMP) was recorded immediately after achieving the whole‐cell configuration. Input resistance (Rin) was calculated from voltage responses to current steps delivered from a holding potential of −70 mV (1 min duration), using linear regression analysis. Neuronal excitability was evaluated by constructing firing frequency–current (F–I) relationships. Action potentials were evoked by stepwise somatic current injections ranging from −40 to 200 pA.

For E/I ratio measurements, the pipette solution contained (in mM): 135 CsMeSO_3_, 10 HEPES, 1 EGTA, 4 MgATP, 0.3 NaGTP, 8 Na_2_‐Phosphocreatine, 3.3 QX314 bromide (adjusted to pH 7.3 with CsOH). Evoked EPSCs and IPSCs were recorded from individual neurons at holding potentials of −70 mV (EPSCs)and 0 mV (IPSCs), respectively, using a bipolar electrode (0.2 ms pulse). The E/I ratio was calculated as the ratio of EPSCs to IPSCs. All data were analyzed using Clampfit 10 software (Molecular Devices, USA).

### 5.14 Luciferase Assays

Upstream 1000 bp of the *Ccl5* promoter fragment was subcloned into the luciferase reporter plasmid. Plasmids of the *Ccl5* promoter and the *Pbrm1*‐overexpression plasmid (OE) were co‐transfected into 293T cells. To determine the motif of PBRM1 binding to the *Ccl5* promoter, the PBRM1‐overexpression vector was co‐transfected into 293T cells together with wild‐type or mutant *Ccl5* promoter plasmid. Cells were collected and lysed using lysis buffer (11402ES60, Yeasen, China) 24 or 48 h following transfection. Then, the luciferase activity was quantified with the Dual‐Luciferase Assay System using a luminometer (BioScan, USA).

### 5.15 Western Blotting Analysis

Equal amounts of denatured protein were resolved by SDS‐PAGE and transferred onto PVDF membranes (IPVH00010, Millipore, USA). Antibodies included rabbit anti‐PBRM1 antibody (1:1000, HPA015629, Sigma–Aldrich, USA), rabbit anti‐H3K18ac (1:1000, A7257, ABclonal, China), rabbit anti‐H3K14ac (1:1000, ab52946, Abcam, UK), rabbit anti‐H3K27ac antibody (1:1000, ab4729, Abcam, UK), mouse anti‐H3K56ac (1:1000, PTM‐162, PTM Bio, China), rabbit anti‐β‐Catenin (1:1000, ab32572, Abcam, UK), rabbit anti‐p‐β‐Catenin (1:1000, AP0579, ABclonal), rabbit anti‐p‐GSK3β (1:1000, ab75814, Abcam, UK), mouse anti‐p‐AKT (1:1000, K000186M, Solarbio, China), rabbit anti‐p‐PI3K (1:1000, AP0854, ABclonal, China), rabbit anti‐CCR5 (1:1000, ER65863, HUABIO, China), mouse anti‐CCR1 (1:500, R&D systems, MAB5986, USA), rabbit anti‐PSD95 (1:1000, ab18258, Abcam, UK). GAPDH (Mouse, 1:10 000, BE0034, Easybio, China) and Tubulin (Mouse, 1:10 000, BE3312, Easybio, China) were used as loading controls. Uncropped blot images are provided in the Supporting Information.

### 5.16 Flow Cytometry

Hippocampus from P15 mice was dissociated using the Papain Dissociation System kit (LK003150, Worthington, USA) after transcardial perfusion. Microglia were isolated by CD11b magnetic bead‐based positive selection (130‐093‐634, Miltenyi Biotec, Germany). Cells were then stained with anti‐mouse TNFα FITC (130‐124‐212, Miltenyi Biotec, Germany) and anti‐mouse IL‐1α PE (130‐104‐530, Miltenyi Biotec, Germany) antibodies for phenotypic analysis. For cells, hMGLs were digested with TrypLE and subsequently stained with anti‐human TNFα APC (502913, Biolegend, USA) and anti‐human IL‐1α PE (500107, Biolegend, USA). FlowJo software (BD, USA) was used to calculate the percentage of positively stained cells within defined cell populations.

### 5.17 Behavioral Tests

Behavioral experiments were performed on 6–8‐week‐old mice. Experimenters were blinded to the genotype during all behavioral assessments. Behavioral analyses were conducted across three experimental conditions with the following sample sizes: WT and *Pbrm1* cKO mice (n = 11–12 per group), temozolomide (TMZ) or vehicle treatment (n = 7–8 per group), and CCR1/5 inhibitor experiments including vehicle‐, BX471‐, and maraviroc‐treated mice (n = 8 per group). Both male and female mice were used, and sexes were balanced across all groups. Littermate controls were included in all behavioral assays. All experiments were recorded and analyzed by the EthoVision XT 14 software (Noldus, The Netherlands).

#### 5.17.1 Open Field Test

Each mouse was placed individually in the center of the arena (40 × 40 × 40 cm) and allowed to explore freely for 10 min. During this period, the distance traveled, the number of entries into the center zone (25%), and the time spent in the center zone were recorded and analyzed.

#### 5.17.2 Elevated Plus Maze Test

The elevated plus maze (EPM) test was conducted according to a previously described protocol [89]. Mice were gently placed in the central platform facing an open arm and allowed to explore freely for 10 min. The time spent in the open arms (40 × 9.5 cm) and closed arms (40 × 9.5 × 9.5 cm) was recorded and analyzed.

#### 5.17.3 Three‐Chamber Social Interaction Test

Social behavior was assessed using a three‐chamber apparatus following a previously published protocol [90]. The apparatus consisted of three equally sized chambers (20 × 40 × 22 cm) connected by small openings (5 × 5 cm) in the dividing walls. During the habituation period, the subject mouse was placed in the central chamber. In the sociability phase, an unfamiliar mouse (Stranger 1) was enclosed in a wire cage in one side chamber, while an empty cage was placed in the opposite chamber. In the social novelty phase, a second unfamiliar mouse (Stranger 2) was introduced into the previously empty cage. Mice were allowed to freely explore the apparatus for 10 min during each phase. The time spent in each chamber and interaction with the unfamiliar mouse and empty cage were recorded and analyzed.

#### 5.17.4 Marble Burying Test

The marble burying test was conducted in standard rodent housing cages. 20 black glass marbles (15 mm in diameter) were placed on the surface of 5 cm‐deep clean corncob bedding. Mice were gently placed into a corner of the cage and allowed to explore freely for 30 min in an undisturbed environment. A picture of each cage was taken, and the number of buried marbles was recorded.

#### 5.17.5 Self‐Grooming Test

Mice were individually placed in a 20 × 20 cm transparent observation chamber and recorded for 30 min. The cumulative duration of self‐grooming behavior was scored offline by an experimenter blinded to genotype.

#### 5.17.6 Morris Water Maze Test

Spatial learning and memory were evaluated using the Morris water maze. The testing apparatus consisted of a circular pool (120 cm diameter) filled with water rendered opaque by non‐toxic white paint. A circular escape platform (13 cm in diameter) was positioned in the target quadrant and submerged 0.5–1 cm below the water surface. Mice were trained for 6 consecutive days, with 4 trials per day. In each trial, animals were released from the pool wall and given up to 60 s to locate the hidden platform, where they were allowed to remain for 15–20 s. The release quadrant was randomized across trials for each mouse and varied among individuals. A probe trial was performed 24 h after the final training session by removing the escape platform. The time spent in the target quadrant and the number of target platform crossings were recorded.

#### 5.17.7 Y Maze Test

The Y‐maze apparatus consisted of three identical arms (40 × 10 × 10 cm) arranged at 120° angles. Mice were placed in the center of the maze facing one of the arms and allowed to freely explore all three arms for 10 min. The spontaneous alternation percentage was recorded.

#### 5.17.8 Novel Object Recognition Test

Recognition memory was assessed using a square open‐field arena (40 × 40 × 40 cm) over two consecutive days, consisting of three phases (habituation, training, and testing), each lasting 10 min. During the habituation period, mice were individually placed in the arena, following the same procedure as the open field test. In the training phase, mice were placed in the arena containing two identical objects. Exploration was defined as directing the nose toward the object at a distance of ≤ 2 cm. After training, mice were returned to their home cages for a 2 h retention interval before the test phase. In the test phase, one familiar object was replaced with a novel object differing in shape and texture, and mice were reintroduced into the arena. The novel object recognition (NOR) index was calculated as the ratio of the exploration time spent on the novel object to the total exploration time of both novel and familiar objects.

### 5.18 Chromatin Immunoprecipitation (ChIP) ‐qPCR Assay

BV2 cells and primary microglia were crosslinked with 1% formaldehyde for 20 min at room temperature, and the reaction was stopped with 0.125 M glycine. After nuclei isolation, chromatin was sonicated to generate ∼150–500 bp DNA fragments. Immunoprecipitation was performed with antibodies against PBRM1 (HPA015629, Sigma, USA), BRG1 (#49360, Cell Signaling Technology, USA), H3K14ac (ab52946, Abcam, UK), and IgG (#2729, Cell Signaling Technology, USA). DNA released from the complexes by Proteinase K digestion was quantified by qPCR with primers targeting specific promoter regions. Relative enrichment was calculated by normalizing ChIP DNA to IgG. Primer sequences were provided in Table S1.

### 5.19 Single‐Cell RNA Sequencing Analysis

#### 5.19.1 Processing of Raw scRNA‐seq Data

Raw sequencing data were processed using Cell Ranger (version 6.0.2, 10× Genomics) with default parameters. Reads were aligned to the mouse reference genome (GRCm38/mm10) and used for gene expression quantification and annotation.

#### 5.19.2 Quality Control

Raw expression matrices from each individual specimen were loaded as Seurat [91] objects (version 4.1.0) in R (version 4.4.0) using the CreateSeuratObject function. For all single‐cell samples, low‐quality cells were excluded according to the following thresholds: (1) detected gene counts between 1 200 and 7 500; (2) mitochondrial gene count percentage below 10%; and (3) the percentage of total counts mapped to hemoglobin genes was below 5%. The resulting filtered matrix comprised 22 517 genes and 22 201 cells.

#### 5.19.3 Cell Clustering and Annotation

WT and cKO datasets were integrated into a single Seurat object for downstream analysis. Data normalization, feature selection, and scaling were performed using the Seurat package. After PCA‐based dimensionality reduction, cell clustering was performed and visualized using UMAP.

Cluster‐enriched marker genes were identified using FindAllMarkers (logfc.threshold = 0.25, only.pos = TRUE, test.use = “wilcox”). Based on canonical markers, 8 cell types were annotated from 35 clusters. Additionally, we isolated and identified subtypes within the Microglial cell type. Fifteen initial clusters were identified. Clusters lacking canonical microglial markers or expressing lineage‐specific markers of other cell types were excluded from downstream analysis, leaving seven microglial subclusters.

The proportions of microglia subtypes in WT and cKO samples were analyzed and visualized as stacked bar plots using ggplot2. Differences in the proportions of microglia subtypes between WT and cKO were assessed using Pearson's chi‐squared test.

#### 5.19.4 DEGs Analysis and GO Enrichment Analysis

DEGs between WT and cKO cells were identified using FindMarkers in each major cell type and microglial subcluster, using criteria of |log_2_FC| > 0 and adjusted *p*‐value < 0.05. GO term enrichment analysis was performed using the clusterProfiler package (version 4.12.0).

#### 5.19.5 Pseudotime Analysis of Microglia

The Monocle3 [92] package was used to construct single‐cell trajectories. The cluster_cells function was applied to cluster microglia subtypes. A trajectory graph was generated using the learn_graph function. Pseudotime values were subsequently assigned to individual cells using the order_cells function to define the position along the trajectory. Cell trajectories were visualized with the plot_cells function.

#### 5.19.6 Analysis of Cell–Cell Communication

To infer, analyze, and visualize differences in cell–cell interactions between WT and cKO, we used the CellChat [93] package (version 1.6.1). Separate CellChat objects were created for WT and cKO groups, and the “Secreted Signaling” database was used for the inference of intercellular communication. Ligand‐receptor interactions with overexpressed signals were identified using the identifyOverExpressedGenes and identifyOverExpressedInteractions functions, followed by computation of communication probabilities with computeCommunProb and pathway‐level aggregation with computeCommunProbPathway. Differences in interaction number and strength between WT and cKO were summarized and visualized using compareInteractions, and interaction differences between cell type pairs were visualized using netVisual_heatmap. To control for differences in cell type abundances between WT and cKO, we set population size = FALSE and performed repeated downsampling (minimum 20 cells per type per group, 50 repeats) and stratified permutation testing. Interaction strength differences between WT and cKO were assessed using a two‐sided permutation test with 200 label permutations. The major sending and receiving cell populations in 2D signaling‐role space were compared with netAnalysis_signalingRole_scatter, and pathway‐specific outgoing signaling roles for each cell type were displayed with netAnalysis_signalingRole_heatmap.

### 5.20 RNA‐seq Analysis

Total RNA was extracted from CD11b^+^ microglia (isolated by magnetic‐activated cell sorting) and amplified by the Smart‐Seq2 protocol. Libraries were prepared after cDNA sonication and sequenced on an Illumina HiSeq platform (PE150, BGI). Raw sequencing quality was assessed using FastQC (v0.11.7), and adapter‐containing reads were removed using FASTX‐Toolkit. DEGs were defined by |log_2_FC| > 0 and adjusted *p*< 0.05. Volcano plots for selected genes and bar plots of GO terms were generated using ggplot2. Heatmaps were generated from the TPM expression matrix using the pheatmap package (v1.34.0).

### 5.21 ATAC‐seq Library Preparation

The ATAC‐seq assay was performed using the Hyperactive ATAC‐Seq Library Prep Kit for Illumina (#TD711‐01, Vazyme, China) following the recommended protocol. Briefly, nuclei were isolated, and transposed DNA fragments were purified with DNA Clean Beads. Libraries were amplified by PCR. After amplification, library size selection was performed with sequential 0.55× and 1× bead‐to‐sample ratios to obtain the desired fragment distribution. Library quality was assessed with a Qubit 3.0 and Bioanalyzer 2100, and qualified libraries were sequenced on an Illumina HiSeq X Ten platform (Annoroad Gene Technology, Beijing).

### 5.22 ATAC‐seq Analysis

ATAC‐seq data were analyzed using an ENCODE‐adapted pipeline. Raw data were assessed with FastQC (v.0.11.7) and aligned to mm10 using Bowtie2 (v2.3.5.1). Low‐quality, mitochondrial, and blacklist‐associated reads were removed, and PCR duplicates were marked. Differential accessibility analysis was performed using DiffBind (v2.14.0). Peaks with increased or decreased chromatin accessibility following PBRM1 deletion were defined based on fold change (FC > 0 or FC < 0) and FDR < 0.05. Peak annotation was performed using ChIPseeker (v.1.22.1) with promoter regions defined as ± 1000 bp from transcription start sites (TSS). GO enrichment was performed with clusterProfiler (v4.18.2). For genome browser visualization, bigWig files (normalized by RPGC) generated using deepTools (v3.3.1) were visualized with Integrative Genomics Viewer (IGV, v2.4.10). Heatmaps and profile plots were created using the “computeMatrix”, “plotHeatmap”, and “plotProfile” functions of deepTools.

### 5.23 Quantification and Statistical Analysis

#### 5.23.1 Cell Quantification

For cell quantification, every 4th serial section was selected from the hippocampal region spanning bregma −1.5 to −3.5 mm. Images were captured using a Zeiss LSM880 confocal microscope with ZEN software, and the number of BrdU^+^ cells co‐labeled with SOX2, DCX, or NeuN in the hippocampus was quantified using ImageJ.

#### 5.23.2 Dendritic Morphology Analysis

For dendritic morphology analysis, 150 µm brain sections containing retrovirus‐labeled neurons were imaged on a Zeiss LSM 880 confocal microscope with a 20× objective. Z‐stack images were acquired at 2 µm intervals and processed with the Sholl analysis plugin in ImageJ.

#### 5.23.3 Statistical Analysis

Statistical analyses were carried out with GraphPad Prism 10 (GraphPad Software). Data distribution was assessed using the Shapiro–Wilk test. For normally distributed data, unpaired two‐tailed Student's *t*‐test or one‐way ANOVA followed by Tukey's post hoc test was applied as appropriate. For non‐normally distributed data, Mann–Whitney *U*‐test or Kruskal–Wallis test followed by Dunn's post hoc test was used for group comparison. The three‐chamber social interaction test was analyzed using two‐way ANOVA followed by Šídák's multiple comparisons test. Sex‐related analyses were performed as described in Tables S2 and S3. Data are shown as mean ± standard error (SEM), and statistical significance was defined as *p*< 0.05.

## Author Contributions

**Yang‐Yang Duan**: writing – original draft, investigation, methodology, formal analysis, data curation. **Lin‐Fei Jiao**: methodology, software, resources. **Pei‐Pei Liu**: methodology, validation, project administration, data curation. **Chun‐Hui Duan**: methodology, project administration. **Xiao Li**: software. **Zi‐Han Zhang**: investigation. **Yue‐Qi Zhang**: methodology, resources. **Xin‐Li Ouyang**: investigation. **Hong‐Zhen Du**: resources, investigation. **Sha Liu**: writing – review and editing. **Yi Tang**: methodology, funding acquisition, project administration. **Zhao‐Qian Teng**: conceptualization, validation, writing – review and editing, supervision. **Chang‐Mei Liu**: conceptualization, writing – review and editing, project administration, supervision, funding acquisition.

## Conflicts of Interest

The authors declare no conflicts of interest.

## Supporting information




**Supporting File**: advs78086‐sup‐0001‐SuppMat.docx.

## Data Availability

All raw sequencing data from this study, including bulk RNA‐seq, ATAC‐seq, and single‐cell RNA‐seq, have been submitted to the Genome Sequence Archive (GSA) at the National Genomics Data Center (NGDC), China National Center for Bioinformation (CNCB), and are available under accession number CRA028463. The dataset can be publicly accessed at https://ngdc.cncb.ac.cn.

## References

[1] M. Sato , N. Nakai , S. Fujima , K. Y. Choe , and T. Takumi , “Social Circuits And Their Dysfunction In Autism Spectrum Disorder,” Molecular Psychiatry 28 (2023): 3194–3206. 10.1038/s41380-023-02201-0 PMC10618103 37612363

[2] Y. Cheng , Z.‐M. Wang , W. Tan , et al., “Partial Loss of Psychiatric Risk Gene Mir137 in Mice Causes Repetitive Behavior And Impairs Sociability And Learning Via Increased Pde10a,” Nature Neuroscience 21 (2018): 1689–1703. 10.1038/s41593-018-0261-7 PMC6261680 30397325

[3] M. Elsabbagh , G. Divan , Y.‐J. Koh , et al., “Global Prevalence Of Autism And Other Pervasive Developmental Disorders,” Autism Research 5 (2012): 160–179. 10.1002/aur.239 PMC3763210 22495912

[4] R. C. M. Philip , M. R. Dauvermann , H. C. Whalley , K. Baynham , S. M. Lawrie , and A. C. Stanfield , “A Systematic Review And Meta‐Analysis of the fMRI Investigation Of Autism Spectrum Disorders,” Neuroscience & Biobehavioral Reviews 36 (2012): 901–942. 10.1016/j.neubiorev.2011.10.008 22101112

[5] M. Ronemus , I. Iossifov , D. Levy , and M. Wigler , “The Role of de novo Mutations In The Genetics Of Autism Spectrum Disorders,” Nature Reviews Genetics 15 (2014): 133–141. 10.1038/nrg3585 24430941

[6] S. J. Sanders , X. He , A. J. Willsey , et al., “Insights Into Autism Spectrum Disorder Genomic Architecture and Biology From 71 Risk Loci,” Neuron 87 (2015): 1215–1233. 10.1016/j.neuron.2015.09.016 PMC4624267 26402605

[7] J. A. S. Vorstman , J. R. Parr , D. Moreno‐De‐Luca , R. J. L. Anney , J. I. Nurnberger Jr , and J. F. Hallmayer , “Autism Genetics: Opportunities And Challenges For Clinical Translation,” Nature Reviews Genetics 18 (2017): 362–376. 10.1038/nrg.2017.4 28260791

[8] J. D. Buxbaum , “Multiple Rare Variants In The Etiology Of Autism Spectrum Disorders,” Dialogues in Clinical Neuroscience 11 (2009): 35–43. 10.31887/DCNS.2009.11.1/jdbuxbaum PMC3181906 19432386

[9] B. J. O'Roak , L. Vives , S. Girirajan , et al., “Sporadic Autism Exomes Reveal A Highly Interconnected Protein Network of de novo Mutations,” Nature 485 (2012): 246–250. 10.1038/nature10989 PMC3350576 22495309

[10] A. S. Nord , W. Roeb , D. E. Dickel , et al., “Reduced Transcript Expression Of Genes Affected By Inherited and de novo CNVs in Autism,” European Journal of Human Genetics 19 (2011): 727–731. 10.1038/ejhg.2011.24 PMC3110052 21448237

[11] B. M. Neale , Y. Kou , L. Liu , et al., “Patterns and Rates Of Exonic de novo Mutations In Autism Spectrum Disorders,” Nature 485 (2012): 242–245. 10.1038/nature11011 PMC3613847 22495311

[12] W. Wenderski , L. Wang , A. Krokhotin , et al., “Loss of the Neural‐Specific BAF Subunit ACTL6B Relieves Repression Of Early Response Genes And Causes Recessive Autism,” Proceedings of the National Academy of Sciences 117 (2020): 10055–10066. 10.1073/pnas.1908238117 PMC7211998 32312822

[13] K. J. Chou , C. H. Yu , Y. T. Yan , P. H. Kuo , and S. J. Tsai , “Association of PBRM1 rs2251219 With Major Depressive Disorder And Biochemical Variables In A Taiwanese Cohort,” Journal of the Chinese Medical Association 88 (2025): 650–654. 10.1097/JCMA.0000000000001245 PMC12637117 40340985

[14] P. J. Ho , J. Kweon , L. A. Blumensaadt , et al., “Multi‐Omics Integration Identifies Cell‐State‐Specific Repression by PBRM1‐PIAS1 Cooperation,” Cell Genomics 4 (2024): 100471. 10.1016/j.xgen.2023.100471 PMC10794847 38190100

[15] V. Waldhauser , T. Baroti , F. Frob , and M. Wegner , “PBAF Subunit Pbrm1 Selectively Influences the Transition From Progenitors to Pre‐Myelinating Cells During Oligodendrocyte Development,” Cells‐Basel 12 (2023): 1556. 10.3390/cells12121556 PMC10296403 37371026

[16] J. R. Lukens and U. B. Eyo , “Microglia and Neurodevelopmental Disorders,” Annual Review of Neuroscience 45 (2022): 425–445. 10.1146/annurev-neuro-110920-023056 PMC10449242 35436413

[17] E. C. Wright‐Jin and D. H. Gutmann , “Microglia as Dynamic Cellular Mediators of Brain Function,” Trends in Molecular Medicine 25 (2019): 967–979. 10.1016/j.molmed.2019.08.013 PMC6829057 31597593

[18] G. Fan , J. Ma , R. Ma , et al., “Microglia Modulate Neurodevelopment in Autism Spectrum Disorder and Schizophrenia,” International Journal of Molecular Sciences 24 (2023): 17297. 10.3390/ijms242417297 PMC10743577 38139124

[19] Z.‐X. Xu , G. H. Kim , J.‐W. Tan , et al., “Elevated Protein Synthesis In Microglia Causes Autism‐Like Synaptic And Behavioral Aberrations,” Nature Communications 11 (2020): 1797. 10.1038/s41467-020-15530-3 PMC7156673 32286273

[20] C. Liu , J. Liu , H. Gong , T. Liu , X. Li , and X. Fan , “Implication of Hippocampal Neurogenesis in Autism Spectrum Disorder: Pathogenesis and Therapeutic Implications,” Current Neuropharmacology 21 (2023): 2266–2282. 10.2174/1570159X21666221220155455 PMC10556385 36545727

[21] S. M. Banker , X. Gu , D. Schiller , and J. H. Foss‐Feig , “Hippocampal Contributions To Social And Cognitive Deficits In Autism Spectrum Disorder,” Trends in Neurosciences 44 (2021): 793–807. 10.1016/j.tins.2021.08.005 PMC8484056 34521563

[22] B. Artegiani , A. Lyubimova , M. Muraro , J. H. van Es , A. van Oudenaarden , and H. Clevers , “A Single‐Cell RNA Sequencing Study Reveals Cellular and Molecular Dynamics of the Hippocampal Neurogenic Niche,” Cell Reports 21 (2017): 3271–3284. 10.1016/j.celrep.2017.11.050 29241552

[23] L. Tan , W. Ma , H. Wu , et al., “Changes in Genome Architecture And Transcriptional Dynamics Progress Independently Of Sensory Experience During Post‐Natal Brain Development,” Cell 184 (2021): 741–758.e17. 10.1016/j.cell.2020.12.032 33484631

[24] Y. Su , Y. Zhou , M. L. Bennett , et al., “A Single‐Cell Transcriptome Atlas Of Glial Diversity In The Human Hippocampus Across The Postnatal Lifespan,” Cell Stem Cell 29 (2022): 1594–1610.e8. 10.1016/j.stem.2022.09.010 PMC9844262 36332572

[25] G. Popova , S. S. Soliman , C. N. Kim , et al., “Human Microglia States Are Conserved Across Experimental Models And Regulate Neural Stem Cell Responses In Chimeric Organoids,” Cell Stem Cell 28 (2021): 2153–2166.e6. 10.1016/j.stem.2021.08.015 PMC8642295 34536354

[26] Q. Li , Z. Cheng , L. Zhou , et al., “Developmental Heterogeneity of Microglia and Brain Myeloid Cells Revealed by Deep Single‐Cell RNA Sequencing,” Neuron 101 (2019): 207–223.e10. 10.1016/j.neuron.2018.12.006 PMC6336504 30606613

[27] J. A. Stogsdill , K. Kim , L. Binan , S. L. Farhi , J. Z. Levin , and P. Arlotta , “Pyramidal Neuron Subtype Diversity Governs Microglia States In The Neocortex,” Nature 608 (2022): 750–756. 10.1038/s41586-022-05056-7 PMC10502800 35948630

[28] T. Masuda , R. Sankowski , O. Staszewski , and M. Prinz , “Microglia Heterogeneity in the Single‐Cell Era,” Cell Reports 30 (2020): 1271–1281. 10.1016/j.celrep.2020.01.010 32023447

[29] F. H. Brennan , Y. Li , C. Wang , et al., “Microglia Coordinate Cellular Interactions During Spinal Cord Repair In Mice,” Nature Communications 13 (2022): 4096. 10.1038/s41467-022-31797-0 PMC9283484 35835751

[30] Y. Li , Z. Li , M. Yang , et al., “Decoding the Temporal And Regional Specification Of Microglia In The Developing Human Brain,” Cell Stem Cell 29 (2022): 620–634.e6. 10.1016/j.stem.2022.02.004 35245443

[31] O. Matcovitch‐Natan , D. R. Winter , A. Giladi , et al., “Microglia Development Follows A Stepwise Program To Regulate Brain Homeostasis,” Science 353 (2016): aad8670. 10.1126/science.aad8670 27338705

[32] W. Cai , L. Su , L. Liao , et al., “PBRM1 Acts as a p53 Lysine‐Acetylation Reader To Suppress Renal Tumor Growth,” Nature Communications 10 (2019): 5800. 10.1038/s41467-019-13608-1 PMC6925188 31863007

[33] L. Liao , N. L. Alicea‐Velázquez , L. Langbein , et al., “High Affinity Binding of H3K14ac Through Collaboration Of Bromodomains 2, 4 and 5 is Critical For The Molecular And Tumor Suppressor Functions of PBRM1,” Molecular Oncology 13 (2019): 811–828. 10.1002/1878-0261.12434 PMC6441893 30585695

[34] J. Liao , J. Ho , M. Burns , E. C. Dykhuizen , and D. C. Hargreaves , “Collaboration Between Distinct SWI/SNF Chromatin Remodeling Complexes Directs Enhancer Selection And Activation Of Macrophage Inflammatory Genes,” Immunity 57 (2024): 1780–1795.e6. 10.1016/j.immuni.2024.05.008 PMC11324393 38843835

[35] J. Yuan , K. Chen , W. Zhang , and Z. Chen , “Structure of Human Chromatin‐Remodelling PBAF Complex Bound To A Nucleosome,” Nature 605 (2022): 166–171. 10.1038/s41586-022-04658-5 35477757

[36] D. Aldinucci , C. Borghese , and N. Casagrande , “The CCL5/CCR5 Axis in Cancer Progression,” Cancers 12 (2020): 1765. 10.3390/cancers12071765 PMC7407580 32630699

[37] M. Huang , J. Jiao , H. Cai , et al., “C‐C Motif Chemokine Ligand 5 Confines Liver Regeneration By Down‐Regulating Reparative Macrophage‐Derived Hepatocyte Growth Factor In A Forkhead Box O 3a–Dependent Manner,” Hepatology 76 (2022): 1706–1722. 10.1002/hep.32458 PMC9790589 35288960

[38] B. P. Festa , F. H. Siddiqi , M. Jimenez‐Sanchez , et al., “Microglial‐to‐Neuronal CCR5 Signaling Regulates Autophagy In Neurodegeneration,” Neuron 111 (2023): 2021–2037.e12. 10.1016/j.neuron.2023.04.006 37105172

[39] A. Ciechanowska and J. Mika , “CC Chemokine Family Members′ Modulation as a Novel Approach for Treating Central Nervous System and Peripheral Nervous System Injury—A Review of Clinical and Experimental Findings,” International Journal of Molecular Sciences 25 (2024): 3788. 10.3390/ijms25073788 PMC11011591 38612597

[40] S. Bolte , S. Girdler , and P. B. Marschik , “The Contribution of Environmental Exposure To The Etiology Of Autism Spectrum Disorder,” Cellular and Molecular Life Sciences 76 (2019): 1275–1297. 10.1007/s00018-018-2988-4 PMC6420889 30570672

[41] D. Bai , B. H. K. Yip , G. C. Windham , et al., “Association of Genetic and Environmental Factors With Autism in a 5‐Country Cohort,” JAMA Psychiatry 76 (2019): 1035–1043. 10.1001/jamapsychiatry.2019.1411 PMC6646998 31314057

[42] M. Li , L. Shen , L. Chen , et al., “Novel Genetic Susceptibility Loci Identified By Family Based Whole Exome Sequencing In Han Chinese Schizophrenia Patients,” Translational Psychiatry 10 (2020): 5. 10.1038/s41398-020-0708-y PMC7026419 32066673

[43] E. G. Porter and E. C. Dykhuizen , “Individual Bromodomains of Polybromo‐1 Contribute to Chromatin Association and Tumor Suppression in Clear Cell Renal Carcinoma,” Journal of Biological Chemistry 292 (2017): 2601–2610. 10.1074/jbc.M116.746875 PMC5314159 28053089

[44] L. B. Su , M. Zhang , F. Ji , et al., “Microglia Homeostasis Mediated by Epigenetic ARID1A Regulates Neural Progenitor Cells Response And Leads To Autism‐Like Behaviors,” Molecular Psychiatry 29, no. 6 (2022): 1595–1609. 10.1038/s41380-022-01703-7 35858990

[45] J. Zhang , P. Rong , L. Zhang , et al., “IL4‐Driven Microglia Modulate Stress Resilience Through BDNF‐Dependent Neurogenesis,” Science Advances 7 (2021): abb9888. 10.1126/sciadv.abb9888 PMC7968840 33731342

[46] V. Fontaine , S. Mohand‐Said , N. Hanoteau , C. Fuchs , K. Pfizenmaier , and U. Eisel , “Neurodegenerative and Neuroprotective Effects Of Tumor Necrosis Factor (TNF) in Retinal Ischemia: Opposite Roles of TNF Receptor 1 and TNF Receptor 2,” The Journal of Neuroscience 22 (2002): RC216–RC216. 10.1523/JNEUROSCI.22-07-j0001.2002 PMC6758303 11917000

[47] E. Courchesne , P. R. Mouton , M. E. Calhoun , et al., “Neuron Number And Size In Prefrontal Cortex Of Children With Autism,” Jama 306 (2011): 2001–2010. 10.1001/jama.2011.1638 22068992

[48] H. Kim , B. Cho , H. Park , et al., “Dormant State Of Quiescent Neural Stem Cells Links Shank3 Mutation To Autism Development,” Molecular Psychiatry 27 (2022): 2751–2765. 10.1038/s41380-022-01563-1 35444258

[49] M. Parellada , M. J. Penzol , L. Pina , et al., “The Neurobiology of Autism Spectrum Disorders,” European Psychiatry 29 (2014): 11–19. 10.1016/j.eurpsy.2013.02.005 24275633

[50] J. Gilbert and H. Y. Man , “Fundamental Elements in Autism: From Neurogenesis and Neurite Growth to Synaptic Plasticity,” Frontiers in Cellular Neuroscience 11 (2017): 359. 10.3389/fncel.2017.00359 PMC5701944 29209173

[51] C. Liu , J. Liu , H. Gong , T. Liu , X. Li , and X. Fan , “Implication of Hippocampal Neurogenesis in Autism Spectrum Disorder: Pathogenesis and Therapeutic Implications,” Current Neuropharmacology 21 (2023): 2266–2282. 10.2174/1570159X21666221220155455 PMC10556385 36545727

[52] L. E. Rexrode , J. Hartley , K. C. Showmaker , et al., “Molecular Profiling Of The Hippocampus Of Children With Autism Spectrum Disorder,” Molecular Psychiatry 29 (2024): 1968–1979. 10.1038/s41380-024-02441-8 PMC11408253 38355786

[53] A. Oguro‐Ando , R. A. Bamford , W. Sital , et al., “Cntn4, A Risk Gene For Neuropsychiatric Disorders, Modulates Hippocampal Synaptic Plasticity And Behavior,” Translational Psychiatry 11 (2021): 106. 10.1038/s41398-021-01223-y PMC7862349 33542194

[54] J. F. Webster and M. A. Cousin , “Comorbidity Of Autism Spectrum Disorders And Anxiety Disorders: Insights From Neuronal Circuitry Studies,” Neuroscience & Biobehavioral Reviews 182 (2026): 106547. 10.1016/j.neubiorev.2026.106547 41494568

[55] Q. L. Xin , I. Feng , G. Y. Yu , and J. Dean , “Stromal Pbrm1 Mediates Chromatin Remodeling Necessary For Embryo Implantation In The Mouse Uterus,” Journal of Clinical Investigation 134 (2024): e174194. 10.1172/JCI174194 PMC10904057 38426493

[56] B. G. Wilson and C. W. Roberts , “SWI/SNF Nucleosome Remodellers and Cancer,” Nature Reviews Cancer 11 (2011): 481–492. 10.1038/nrc3068 21654818

[57] X. Yao , J. H. Hong , A. M. Nargund , et al., “PBRM1‐Deficient PBAF Complexes Target Aberrant Genomic Loci To Activate the NF‐κB Pathway In Clear Cell Renal Cell Carcinoma,” Nature Cell Biology 25 (2023): 765–777. 10.1038/s41556-023-01122-y 37095322

[58] X.‐D. Liu , W. Kong , C. B. Peterson , et al., “PBRM1 Loss Defines A Nonimmunogenic Tumor Phenotype Associated With Checkpoint Inhibitor Resistance In Renal Carcinoma,” Nature Communications 11 (2020): 2135. 10.1038/s41467-020-15959-6 PMC7195420 32358509

[59] M. Hagiwara , A. Fushimi , A. Bhattacharya , et al., “MUC1‐C Integrates Type II Interferon And Chromatin Remodeling Pathways In Immunosuppression Of Prostate Cancer,” Oncoimmunology 11 (2022): 2029298. 10.1080/2162402X.2022.2029298 PMC8812775 35127252

[60] D. Pan , A. Kobayashi , P. Jiang , et al., “A Major Chromatin Regulator Determines Resistance Of Tumor Cells to T Cell–Mediated Killing,” Science 359 (2018): 770–775. 10.1126/science.aao1710 PMC5953516 29301958

[61] A. L. Wurster , P. Precht , K. G. Becker , et al., “IL‐10 Transcription Is Negatively Regulated by BAF180, a Component of the SWI/SNF Chromatin Remodeling Enzyme,” BMC Immunology 13 (2012): 9. 10.1186/1471-2172-13-9 PMC3313858 22336179

[62] W. Cai , L. Su , and H. Yang , “PBRM1 Suppresses Tumor Growth As A Novel p53 Acetylation Reader,” Molecular & Cellular Oncology 7 (2020): 1729680. 10.1080/23723556.2020.1729680 PMC7199739 32391422

[63] B. Wang , Y. Qin , Q. Wu , et al., “mTOR Signaling Pathway Regulates the Release of Proinflammatory Molecule CCL5 Implicated in the Pathogenesis of Autism Spectrum Disorder,” Frontiers in Immunology 13 (2022): 818518. 10.3389/fimmu.2022.818518 PMC9002353 35422816

[64] A. Ozaslan , E. Guney , O. Gulbahar , D. Buyuktaskin , and B. I. Arslan , “Increased Serum Level of CCL5 in Children With Attention‑Deficit/Hyperactivity Disorder: First Results About Serum Chemokines,” Clinical Psychopharmacology and Neuroscience 20 (2022): 109–117. 10.9758/cpn.2022.20.1.109 PMC8813316 35078953

[65] M. L. Estes and A. K. McAllister , “Immune Mediators in the Brain And Peripheral Tissues In Autism Spectrum Disorder,” Nature Reviews Neuroscience 16 (2015): 469–486. 10.1038/nrn3978 PMC5650494 26189694

[66] J. Zhao , K. An , Z. Mao , et al., “CCL5 Promotes LFA‐1 Expression in Th17 Cells And Induces LCK and ZAP70 Activation In A Mouse Model of Parkinson's Disease,” Frontiers in Aging Neuroscience 15 (2023): 1250685. 10.3389/fnagi.2023.1250685 PMC10655117 38020765

[67] M. F. Lanfranco , I. Mocchetti , M. P. Burns , and S. Villapol , “Glial‐ and Neuronal‐Specific Expression of CCL5 mRNA in the Rat Brain,” Frontiers in Neuroanatomy 11 (2017): 137. 10.3389/fnana.2017.00137 PMC5770405 29375328

[68] R. Ajoy , Y.‐C. Lo , M.‐H. Ho , et al., “CCL5 Promotion Of Bioenergy Metabolism Is Crucial For Hippocampal Synapse Complex And Memory Formation,” Molecular Psychiatry 26 (2021): 6451–6468. 10.1038/s41380-021-01103-3 PMC8760051 33931731

[69] M.‐H. Ho , Y.‐J. Tsai , C.‐Y. Chen , et al., “CCL5 is Essential for Axonogenesis And Neuronal Restoration After Brain Injury,” Journal of Biomedical Science 31 (2024): 91. 10.1186/s12929-024-01083-w PMC11406852 39285280

[70] S. Wegener , A. Buschler , A. V. Stempel , et al., “Defective Synapse Maturation and Enhanced Synaptic Plasticity in Shank2 Deltaex7(‐/‐) Mice,” eNeuro 5 (2018): 0398‐17. 10.1523/ENEURO.0398-17.2018 PMC6049608 30023428

[71] M. Phillips and L. Pozzo‐Miller , “Dendritic Spine Dysgenesis In Autism Related Disorders,” Neuroscience Letters 601 (2015): 30–40. 10.1016/j.neulet.2015.01.011 PMC4496332 25578949

[72] R. T. Ash , J. Park , B. Suter , H. Y. Zoghbi , and S. M. Smirnakis , “Excessive Formation and Stabilization of Dendritic Spine Clusters in the MECP2‐Duplication Syndrome Mouse Model of Autism,” eNeuro 8 (2021): 0282‐20. 10.1523/ENEURO.0282-20.2020 PMC7877475 33168618

[73] P. Licznerski , H.‐A. Park , H. Rolyan , et al., “ATP Synthase c‐Subunit Leak Causes Aberrant Cellular Metabolism in Fragile X Syndrome,” Cell 182 (2020): 1170–1185.e9. 10.1016/j.cell.2020.07.008 PMC7484101 32795412

[74] P. Chen , Y.‐H. Jiang , S.‐Q. Xue , et al., “Astrocytic CCL5 Orchestrates CCR5‐Positive Neuronal Necroptosis In Subarachnoid Hemorrhage,” Journal of Neuroinflammation 23 (2026): 81. 10.1186/s12974-026-03714-5 PMC12973912 41639749

[75] A. Repici , A. P. Capra , A. Hasan , et al., “Novel Findings on CCR1 Receptor in CNS Disorders: A Pathogenic Marker Useful in Controlling Neuroimmune and Neuroinflammatory Mechanisms in Parkinson's Disease,” International Journal of Molecular Sciences 25 (2024): 4337. 10.3390/ijms25084337 PMC11050472 38673922

[76] L. C. Soares , N. Huang , H. Bernhardova , et al., “Galectin‐3 Induces Neurodevelopmental Apical‐Basal Polarity And Regulates Gyrification,” Science Advances 11 (2025): adt5859. 10.1126/sciadv.adt5859 PMC12407091 40901969

[77] I. Srejovic , D. Selakovic , N. Jovicic , V. Jakovljevic , M. L. Lukic , and G. Rosic , “Galectin‐3: Roles in Neurodevelopment, Neuroinflammation, and Behavior,” Biomolecules 10 (2020): 798. 10.3390/biom10050798 PMC7277476 32455781

[78] S. Ishibashi , T. Kuroiwa , M. Sakaguchi , et al., “Galectin‐1 Regulates Neurogenesis In The Subventricular Zone And Promotes Functional Recovery After Stroke,” Experimental Neurology 207 (2007): 302–313. 10.1016/j.expneurol.2007.06.024 17706645

[79] K. Zhang , Q. Zhang , J. Deng , et al., “ALK5 Signaling Pathway Mediates Neurogenesis And Functional Recovery After Cerebral Ischemia/Reperfusion In Rats via Gadd45b,” Cell Death & Disease 10 (2019): 360. 10.1038/s41419-019-1596-z PMC6494915 31043581

[80] J. L. Mignone , V. Kukekov , A. S. Chiang , D. Steindler , and G. Enikolopov , “Neural Stem And Progenitor Cells In Nestin‐GFP Transgenic Mice,” Journal of Comparative Neurology 469 (2004): 311–324. 10.1002/cne.10964 14730584

[81] L. Cong , F. A. Ran , D. Cox , et al., “Multiplex Genome Engineering Using CRISPR/Cas Systems,” Science 339 (2013): 819–823. 10.1126/science.1231143 PMC3795411 23287718

[82] Y.‐J. Xu , P.‐P. Liu , Z.‐Z. Yan , et al., “KW‐2449 and VPA Exert Therapeutic Effects On Human Neurons And Cerebral Organoids Derived From MECP2‐Null hESCs,” Stem Cell Research & Therapy 13 (2022): 534. 10.1186/s13287-022-03216-0 PMC9795779 36575558

[83] J.‐P. Ke , B.‐D. He , M.‐L. Gong , et al., “Loss of Microglial Arid1a Exacerbates Microglial Scar Formation Via Elevated CCL5 After Traumatic Brain Injury,” Cell Communication and Signaling 22 (2024): 467. 10.1186/s12964-024-01852-y PMC11443815 39350161

[84] C. Liu , Z.‐Q. Teng , N. J. Santistevan , et al., “Epigenetic Regulation of miR‐184 by MBD1 Governs Neural Stem Cell Proliferation And Differentiation,” Cell Stem Cell 6 (2010): 433–444. 10.1016/j.stem.2010.02.017 PMC2867837 20452318

[85] P.‐P. Liu , S.‐P. Lu , X. Li , et al., “Abnormal Chromatin Remodeling Caused by ARID1A Deletion Leads To Malformation Of The Dentate Gyrus,” Cell Death & Differentiation 30 (2023): 2187–2199. 10.1038/s41418-023-01199-w PMC10483045 37543710

[86] A. Carrard , F. Cassé , C. Carron , et al., “Role of Adult Hippocampal Neurogenesis In The Antidepressant Actions Of Lactate,” Molecular Psychiatry 26 (2021): 6723–6735. 10.1038/s41380-021-01122-0 PMC8760055 33990772

[87] J. Wang , Y. Cui , Z. Yu , et al., “Brain Endothelial Cells Maintain Lactate Homeostasis and Control Adult Hippocampal Neurogenesis,” Cell Stem Cell 25 (2019): 754–767.e9. 10.1016/j.stem.2019.09.009 31761722

[88] A. Sahay , K. N. Scobie , A. S. Hill , et al., “Increasing Adult Hippocampal Neurogenesis Is Sufficient To Improve Pattern Separation,” Nature 472 (2011): 466–470. 10.1038/nature09817 PMC3084370 21460835

[89] M. Gong , R. Shi , Y. Liu , et al., “Abnormal Microglial Polarization Induced by Arid1a Deletion Leads To Neuronal Differentiation Deficits,” Cell Proliferation 55 (2022): 13314. 10.1111/cpr.13314 PMC9628249 35854653

[90] B. Rein , K. Ma , and Z. Yan , “A Standardized Social Preference Protocol For Measuring Social Deficits In Mouse Models Of Autism,” Nature Protocols 15 (2020): 3464–3477. 10.1038/s41596-020-0382-9 PMC8103520 32895524

[91] Y. Hao , S. Hao , E. Andersen‐Nissen , et al., “Integrated Analysis Of Multimodal Single‐Cell Data,” Cell 184 (2021): 3573–3587.e29. 10.1016/j.cell.2021.04.048 PMC8238499 34062119

[92] C. Trapnell , D. Cacchiarelli , J. Grimsby , et al., “The Dynamics And Regulators Of Cell Fate Decisions Are Revealed By Pseudotemporal Ordering Of Single Cells,” Nature Biotechnology 32 (2014): 381–386. 10.1038/nbt.2859 PMC4122333 24658644

[93] S. Jin , C. F. Guerrero‐Juarez , L. Zhang , et al., “Inference and Analysis of Cell‐Cell Communication Using CellChat,” Nature Communications 12 (2021): 1088. 10.1038/s41467-021-21246-9 PMC7889871 33597522

