# Supplementary material for: PBRM1 Deficiency in Microglia Leads to Excessive Neurogenesis and Autism‐Like Behaviors via the CCL5‐CCR1/5‐β‐catenin Signaling Axis
Source: Adv Sci (Weinh). 2026 Oct 18:e78086. Online ahead of print. doi: 10.1002/advs.78086 (PMC13676817; doi:10.1002/advs.78086)
Supplement: Supplementary file 1 [file ADVS-9999-e78086-s001.docx]

**Supporting information**

**PBRM1 deficiency in microglia leads to excessive neurogenesis and autism-like behaviors via the CCL5-CCR1/5-β-catenin signaling axis**

Yang-Yang Duan,^1,2,3, *^ Lin-Fei Jiao,^1,2,3, *^ Pei-Pei Liu,^1,2,3^ Chun-Hui Duan,^1,2,3^ Xiao Li,^1,2,3^ Zi-Han Zhang,^1,2,3^ Yue-Qi Zhang,^1,2,3^ Xin-Li Ouyang,^1,2,3^ Hong-Zhen Du,^1,2,3^ Sha Liu,^4^ Yi Tang,^5, #^ Zhao-Qian Teng ^1,2,3 #^ Chang-Mei Liu^1,2,3 #^

**Figure S1. Identification of PBRM1 Knockout in Microglia**

A-B. Representative confocal images (A) and quantification (B) of PBRM1 immunostaining in primary microglia, neurons, and astrocytes from WT and *Pbrm1* cKO mice. Scale bar, 40 μm. Mean ± SEM. ns, no significant, ***P* < 0.01 by Student’s t test. n = 3 independent experiments. Males (circles) and females (squares) are indicated.

C. Western blot analysis of isolated primary microglia confirmed lack of PBRM1 protein expression in *Pbrm1* cKO mice. Mean ± SEM. *****P* < 0.0001 by Student’s t test. n = 3 mice per group. Males (circles) and females (squares) are indicated.

D. Representative confocal images showing DAPI (blue), IBA1 (red), and PBRM1 (green) staining in the hippocampus. Scale bars, 50 μm.

**Figure S2. Loss of Microglial PBRM1 Does Not Affect Spatial Learning and Working Memory but Impairs Recognition Memory**

A. Escape latency to reach the platform during acquisition training in the Morris water maze. n = 11-13 mice per group.

B-C. Number of target crossings (B) and time spent in the target quadrant (C) during the probe trial of the Morris water maze test. *Pbrm1* cKO mice showed no significant differences in the number of target crossings and time spent in the target quadrant compared with WT mice. Mean ± SEM. ns, no significant by Mann–Whitney test. n = 11-13 mice per group. Males (circles) and females (squares) are indicated.

D. *Pbrm1* cKO mice showed no significant difference in spontaneous alteration compared with WT mice in the Y maze test. Mean ± SEM. ns, no significant by Mann–Whitney test. n = 12 mice per group. Males (circles) and females (squares) are indicated.

E. Quantification of object recognition index in the novel object recognition test. The *Pbrm1* cKO mice showed reduced preference for the novel object. Mean ± SEM. **P* < 0.05 by Student’s t test. n = 7-8 mice per group. Males (circles) and females (squares) are indicated.

**Figure S3. Single-cell Transcriptomic Analyses of WT and *Pbrm1* cKO Hippocampus**

A. Violin plots showing the number of genes (nFeature_RNA) and reads (nCount_RNA) detected in each cell, as well as the percentage of mitochondrial (percent.mt) and haemoglobin genes per cell after filtering and quality control.

B. Violin plots depicting cell types using established marker genes.

C. UMAP plot showing 22,201 individual cells categorized into eight cell clusters, including microglia (MG), astrocytes (AST), neural stem cells (NSC), oligodendrocytes (OLIG), neurons, endothelial cells, and ependymal cells.

**Figure S4. Remolding Neuroimmune Environment in *Pbrm1* cKO Hippocampus**

A. Bar plots showing the strength (left) and number (right) of cell-cell interactions in the hippocampus of P15 WT versus cKO mice.

B. CellChat analysis among microglia, neurons, and NSPCs. Heatmaps displaying the differential strength (left) and number (right) of interactions in the hippocampus of P15 WT versus cKO mice.

C. Representative confocal images and quantification of IBA1 (microglia), SOX2 (gray), Nestin-GFP (NSPCs) staining in the hippocampus. The cKO mice had an increased ratio of microglia to NSPCs. Scale bars, 20 μm. Mean ± SEM. **P* < 0.05 by Student’s t test. n = 6 mice per group. Males (circles) and females (squares) are indicated.

D-F. Gene Ontology (GO) terms of NSC (D), neuron (E) and astrocyte (F) of scRNA-seq.

**Figure S5. *Pbrm1* cKO Microglia Promote Neurogenesis in *vivo* and in *vitro***

A. Confocal images of DAPI (blue) and BrdU (red), BrdU (red) and DCX (green), BrdU (red) and NeuN (green) immunostaining. Scale bars, 20 μm.

B. Quantification analysis of the number of BrdU^+^ NSPCs, BrdU^+^DCX^+^ immature neurons and BrdU^+^NeuN^+^ mature neurons. The cKO mice had an increased number of BrdU^+^ NSPCs, BrdU^+^DCX^+^ immature neurons, BrdU^+^NeuN^+^ mature neurons. Mean ± SEM. **P* < 0.05 by Student’s t test. n = 3-4 mice per group. Males (circles) and females (squares) are indicated.

C. Confocal images of DAPI (blue), BrdU (red), SOX2 (gray), and Nestin-GFP (green), DCX (green) and BrdU (red), NeuN (green) and BrdU (red) immunostaining in SVZ and olfactory bulb. Scale bars, 50 μm.

D. Quantification analysis of the number of BrdU^+^SOX2^+^Nestin-GFP^+^ NSPCs, BrdU^+^DCX^+^ immature neurons, BrdU^+^NeuN^+^ mature neurons. The cKO mice had an increased number of BrdU^+^SOX2^+^ Nestin-GFP^+^ NSPCs, BrdU^+^DCX^+^ immature neurons, and BrdU^+^NeuN^+^ mature neurons. Mean ± SEM. **P* < 0.05 by Student’s t test. n = 4 mice per group. Males (circles) and females (squares) are indicated.

E-F**.** Representative images (E) and quantification (F) of BrdU (red) and Nestin (green) immunostaining of the proliferation of NSPCs which were indirectly co-cultured with WT or *Pbrm1* cKO microglia in a Transwell system. Scale bars, 40 μm. Mean ± SEM. ***P* < 0.01 by Student’s t test. n = 3 independent experiments. Males (circles) and females (squares) are indicated.

G-H. Representative images (G) and quantification (H) of Tuj1 (red) and GFAP (green) immunostaining of the differentiation of NSPCs which were indirectly co-cultured with WT or *Pbrm1* cKO microglia in a Transwell system. Scale bars, 40 μm. Mean ± SEM. **P* < 0.05 by Student’s t test. n = 3-4 independent experiments. Males (circles) and females (squares) are indicated.

**Figure S6. Identity of hESC-derived Human Microglia-like Cells Used in Co-cultured Organoid**

A. Western blotting showed the expression levels of OCT3/4 and NANOG of hESCs and *PBRM1* KO hESCs. Mean ± SEM. ns, no significant by Student’s t test. n = 3 independent experiments.

B. Quantitative RT-PCR analysis showing the relative mRNA levels of *IL1A*, *TNF, P2RY12* and *TMEM119* in control and *PBRM1* KO hMGLs. Compared to controls, *PBRM1* KO hMGLs displayed significantly lower expression of *P2RY12* and *TMEM119*, but higher expression of *TNF* and *IL1A*. Mean ± SEM. ***P* < 0.01, ****P* < 0.001, *****P* < 0.0001 by Student’s t test. n = 3 independent experiments.

C. Representative flow cytometry gating strategy for sorting IL-1α^+^TNFα^+^ hMGLs differentiated from control and *PBRM1* KO H9 hESC lines.

D. Quantification of the percentage of IL-1α^+^TNFα^+^ hMGLs differentiated from control and *PBRM1* KO H9 hESC lines. Mean ± SEM. ****P* < 0.001 by Student’s t test. n = 5-6 independent experiments.

**Figure S7. Increased Synaptic Density and Excitatory/Inhibitory Imbalance in *Pbrm1* cKO mice**

A. Left, representative image of an RFP-labeled newborn granule neuron in the dentate gyrus (DG) used for whole-cell patch-clamp recordings. Scale bar, 10 μm. Right, representative action potentials recorded from RFP-labeled newborn granule neurons in response to −40 pA, 0 pA, and +140 pA current injections. Scale bars, 20 mV, 200 ms.

B. Quantification of action potential (AP) frequency in RFP-labeled newborn granule neurons from WT and cKO mice. No significant difference was observed between groups. Mean ± SEM. ns, no significance by two-way ANOVA. n = 14 neurons from 7 WT mice, n = 14 neurons from 5 cKO mice.

C. Resting membrane potential of RFP-labeled newborn granule neurons from WT and cKO mice. Mean ± SEM. ns, no significance by Student’s t test. n = 14 neurons from 7 WT mice, n = 14 neurons from 5 cKO mice.

D. Input resistance of RFP-labeled newborn granule neurons from WT and cKO mice. Mean ± SEM. ns, no significance by Mann–Whitney test. n = 14 neurons from 7 WT mice, n = 14 neurons from 5 cKO mice.

E. Representative images of Western blotting for PSD95 in hippocampal tissues from P15 mice.

F. Quantification of the expression levels of PSD95 in hippocampal tissues from P15 mice. Mean ± SEM. ****P* < 0.001 by Student’s t test. n = 4 mice per group. Males (circles) and females (squares) are indicated.

G. Representative confocal images of GFP in WT and *Pbrm1* cKO mice at P15. Scale bars, 5 μm.

H. Quantification of spine density in WT and *Pbrm1* cKO mice. The cKO mice had significantly increased dendritic spine density. Mean ± SEM. *****P* < 0.0001 by Mann–Whitney test. n = 41 synapses from 6 WT mice, n = 43 synapses from 6 cKO mice. Males (circles) and females (squares) are indicated.

I. Representative traces of EPSC and IPSC in neurons in DG region of WT and cKO mice. Scale bars, 50 pA, 50 ms.

J. Quantification of EPSC/IPSC ratio in neurons in DG region of WT and cKO mice. The *Pbrm1* cKO mice showed significantly increased E/I ratio compared with WT. Mean ± SEM. **P* < 0.05 by two-way ANOVA. n = 13-14 neurons from 3-4 mice per group.

**Figure S8. scRNA-seq and Flow Cytometry Analysis of Microglial Clusters**

A. Violin plots showing the expression of marker genes in microglial subclusters.

B. Dot plot showing Gene Ontology (GO) terms of microglial subclusters.

C. Bar plots showing the strength (left) and number (right) of cell-cell interactions in microglia and NSCs of P15 WT and *Pbrm1* cKO mice.

D. Heatmaps displaying the differential strength (left) and number (right) of interactions in the cell-cell communication network among different microglial clusters and NSCs.

E**.** Scatter plots showing dominant senders (sources) and receivers (targets) in a two-dimensional space for WT (left) and *Pbrm1* cKO (right). Outgoing and incoming signaling strengths are shown on the x- and y-axes, respectively.

F. Heatmaps showing outgoing signaling from all microglial subtypes and neural stem cells in WT (left) and *Pbrm1* cKO (right). The top bar plot summarizes the total signaling output of each cell group, and the side bar plot summarizes the overall signaling strength of each pathway.

G. Representative flow cytometry gating strategy for sorting IL-1α^+^, TNF-α^+^ primary microglia after 10h LPS treatment.

**Figure S9. Bulk RNA-seq Analysis of Microglia**

A. GO analysis of biological processes related to the downregulated and upregulated genes in the *Pbrm1* cKO microglia by RNA-seq.

**Figure S10. ATAC-seq Analysis of Microglia and ChIP-seq Analysis of Public Data**

A. Heatmap of ATAC-seq signals at transcription start sites (TSS) in WT (left) and *Pbrm1* cKO microglia (right).

B. Genomic distribution of WT and cKO up-regulated peaks.

C. Gene Ontology (GO) biological process analysis of genes with increased chromatin accessibility in *Pbrm1* cKO microglia compared to WT.

D. UCSC genome browser tracks showing representative ATAC-seq signal profiles in WT and *Pbrm1* cKO microglia across several genomic loci.

**Figure S11. CCL5 Promotes Neurogenesis by Activating β-catenin Pathway**

A. Western blotting showed the expression levels of CCR1 and CCR5 of primary NSPCs (P7) in WT and cKO mice. Mean ± SEM. ns, no significant by Student’s t test. n = 4 mice per group. Males (circles) and females (squares) are indicated.

B. Representative images and quantification of Nestin (green) and BrdU (red) immunostaining of NSPCs treated with exogenous CCL5. The results showed that the exogenous CCL5 treatment group had more proliferating NSPCs. Scale bar, 40 μm. Mean ± SEM. **P* < 0.05 by Student’s t test. n = 3 independent experiments.

C. Representative images and quantification of GFAP (green) and Tuj1 (red) immunostaining of NSPCs treated with exogenous CCL5. NSPCs with the treatment of exogenous CCL5 differentiated into a significantly higher number of Tuj1^+^ cells but a lower number of GFAP^+^ cells. Scale bar, 40 μm. Mean ± SEM. **P* < 0.05 by Student’s t test. n = 3 independent experiments.

D. Western blotting showed the expression levels of β-catenin, p-β-catenin, p-GSK3β and p-AKT in NSPCs treated with exogenous CCL5.

E. Quantifications of the expression levels of β-catenin, p-β-catenin, p-GSK3β, p-AKT in NSPCs treated with exogenous CCL5. CCL5 increased the expression levels of β-catenin, p-β-catenin and p-AKT in NSPCs. Mean ± SEM. ns, no significant, **P* < 0.05, ***P* < 0.01 by one-way ANOVA. n = 3-4 independent experiments.

F-G. Representative confocal images (F) and quantifications (G) of DAPI (blue), BrdU (red), DCX (green) or NeuN (green) immunostaining of hippocampal tissues from CCL5-treated WT mice. Scale bars, 20 μm. The administration of CCL5 increased the number of DCX^+^BrdU^+^ immature neurons and NeuN^+^BrdU^+^ mature neurons in WT mice. Mean ± SEM. ***P* < 0.01 by Student’s t test. n = 4-5 mice per group. Males (circles) and females (squares) are indicated.

**Figure S12. Blockade of CCL5 Rescues *Pbrm1* cKO Microglia-induced Excessive Neurogenesis**

A. Representative images and quantification of Nestin (green) and BrdU (red) immunostaining of the proliferation of NSPCs which were co-cultured with *Pbrm1* cKO microglia and treated with IgG or CCL5 antibody. Scale bar, 40 μm. Mean ± SEM. ***P* < 0.01 by one-way ANOVA. n = 3 independent experiments. Males (circles) and females (squares) are indicated.

B. Representative images and quantification of GFAP (green) and Tuj1 (red) immunostaining of the differentiation of NSPCs which were co-cultured with *Pbrm1* cKO microglia and treated with IgG or CCL5 antibody. Scale bar, 40 μm. Mean ± SEM. ***P* < 0.01, ****P* < 0.001, *****P* < 0.0001 by one-way ANOVA. n = 3-4 independent experiments. Males (circles) and females (squares) are indicated.

C. Quantitative RT-PCR analysis showing the relative mRNA levels of *Pbrm1, Ccl5* in WT and dcKO microglia. Compared to WT, dcKO microglia displayed significantly lower expression of *Pbrm1* and *Ccl5*. Mean ± SEM. ****P* < 0.001. *****P* < 0.0001 by Student’s t test. n = 3 mice per group. Males (circles) and females (squares) are indicated.

**Figure S13. ChIP-seq Analysis of H3K14ac Occupancy at the *Ccl5* Promoter Region**

A. ChIP-seq analysis of H3K14ac occupancy at *Ccl5* locus. Mouse hepatocyte (GSM5170976, mm10) shows clear H3K14ac enrichment at *Ccl5* promoter. Human cancer cell line (GSM4623483, hg19) and MCF-7 cells (GSM8658927, hg19) show no significant H3K14ac enrichment at *Ccl5* promoter. Human HEK293T cells (GSM8637606, hg38) show H3K14ac enrichment at *Ccl5* promoter. Continuous read coverage is shown as bigWig tracks, and gene coordinates for each reference genome are indicated.

**Figure S14. Pharmacological Inhibition of CCR1/CCR5 Rescues Neurogenesis in *Pbrm1* cKO Mice**

A. Western blotting showed the expression levels of β-catenin, p-β-catenin and p-AKT in NSPCs treated with BX471 or maraviroc.

B. Quantifications of the expression levels of β-catenin, p-β-catenin, and p-AKT in NSPCs treated with BX471 or maraviroc. Individual addition of CCR1 or CCR5 did not decrease the expression levels of β-catenin, p-β-catenin and p-AKT in NSPCs which were indirectly co-cultured with *Pbrm1* cKO microglia. Mean ± SEM. ns, no significant, **P* < 0.05, ***P* < 0.01 by one-way ANOVA or Kruskal–Wallis test. n = 3 independent experiments.

C-D. Representative confocal images (C) and quantifications (D) of Nestin-GFP (green) and BrdU (red), BrdU (red) and DCX (green), BrdU (red) and NeuN (green) immunostaining of hippocampal tissues from CCR1/CCR5 antagonist-treated *Pbrm1* cKO mice. Scale bars, 20 μm. The application of maraviroc and BX471 ameliorated the increased number of Nestin-GFP^+^BrdU^+^ NSPCs, DCX^+^BrdU^+^ immature neurons and NeuN^+^BrdU^+^ mature neurons in *Pbrm1* cKO mice. Mean ± SEM. **P* < 0.05, ***P* < 0.01, ****P* < 0.001 by one-way ANOVA or Kruskal–Wallis test. n = 4 mice per group. Males (circles) and females (squares) are indicated.

**Figure S15. ChIP-seq Analysis of BRG1 Occupancy at the *Ccl5* Promoter Region**

A. ChIP-seq analysis of BRG1 occupancy at the *Ccl5* locus. BRG1 (SMARCA4) occupancy at the *Ccl5* promoter was examined in human cancer cell lines (GSM4623477, hg19), fetal liver (GSM5593419, GSM5593420, mm10), P0 mouse retina (GSM1915951, mm9). Signal tracks represent continuous read coverage (bigWig) or discrete peaks (narrowPeak). No BRG1 enrichment was detected at the *Ccl5* promoter.

Table S1 The sequence of primers was used in this study, related to STAR Methods

| PCR-amplified primers | | |
| --- | --- | --- |
| Gene | Forward | Reverse |
| *CCL5* | ATCCCAACTCTGTCATTCATTT | CTTTATAGGGCCAGTTGAGGGG |
| *PBRM1*-gRNA-1 | GTCAACTCAGTTGTTTGAAAGGA | AAACTGTTCATCCTTATAGTCTCC |
| *PBRM1*-gRNA-2 | CACCGCATTAGGGCACCAAAGCGA | AAACTCGCTTTGGTGCCCTAATGC |
| RT-PCR primers | | |
| *β-Actin* | TGCACCACCAACTGCTTA | GGATGCAGGGATGATGTTC |
| *P2ry12* | CATTGACCGCTACCTGAAGACC | GCCTCCTGTTGGTGAGAATCATG |
| *Tmem119* | ACTACCCATCCTCGTTCCCTGA | TAGCAGCCAGAATGTCAGCCTG |
| *Tnf-α* | ACGGCATGGATCTCAAAGAC | GTGGGTGAGGAGCACGTAGT |
| *Ccl5-1* | ACGTCAAGGAGTATTTCTACAC | GATGTATTCTTGAACCCACT |
| *Ccl5-2* | TGGTATTCCAAAAGCCCTATCTGA | GCCAGCAAGCACCCTATTATATTC |
| *Ccl12* | GTCCTCAAGGTATTGGCTGGA | GGGTCAGCACAGATCTCCTT |
| *TMEM119* | AGTCCTGTACGCCAAGGAAC | GCAGCAACAGAAGGATGAGG |
| *P2RY12* | AAGAGCACTCAAGACTTTAC | GGGTTTGAATGTATCCAGTAAG |
| *IL1A* | AGATGCCTGAGATACCCAAAACC | CCAAGCACACCCAGTAGTCT |
| *TNF* | CCTCTCTCTAATCAGCCCTCTG | GAGGACCTGGGAGTAGATGAG |
| ChIP-qPCR primers | | |
| *Ccl5-1* | GATGGACATAGAGGACACAACT | TATCAGAAACAGACCACACTTG |
| *Ccl5-2* | GCACACACACACACACACACAC | TTGTCTGTCTGTCTGTCTGTTT |

Table S2 Three-way ANOVA analysis of sex- and genotype-related effects in the three-chamber social behavior test

| Sociability | | | | | | |
| --- | --- | --- | --- | --- | --- | --- |
| Genotype | Sex | n | Mean ± SEM (E) | Mean ± SEM (S1) | Three-way ANOVA | |
|  |  |  |  |  | Factor | Significance |
| WT | Male | 9 | 70.4 ± 18.4 | 207.9 ± 37.4 | Genotype × chamber | **** |
| WT | Female | 8 | 73.0 ± 18.4 | 212.2 ± 27.9 | Sex × chamber | ns |
| cKO | Male | 9 | 171.1 ± 22.2 | 104.8 ± 20.7 | Genotype × Sex × chamber | ns |
| cKO | Female | 8 | 89.8 ± 18.6 | 121.7 ± 22.4 |  |  |
| Social novelty | | | | | | |
| Genotype | Sex | n | Mean ± SEM (S1) | Mean ± SEM (S2) | Three-way ANOVA | |
|  |  |  |  |  | Factor | Significance |
| WT | Male | 9 | 99.6 ± 14.4 | 206.1 ± 37.4 | Genotype × chamber | *** |
| WT | Female | 8 | 133.3 ± 31.8 | 261.0 ± 34.5 | Sex × chamber | ns |
| cKO | Male | 9 | 119.9 ± 15.5 | 98.1 ± 7.2 | Genotype × Sex × chamber | ns |
| cKO | Female | 8 | 172.5 ± 49.2 | 126.7 ± 23.5 |  |  |

Table S3 Two-way ANOVA analysis of sex- and genotype-related effects on hippocampal neurogenesis in the dentate gyrus

| BrdU^+^NestinGFP^+^SOX2^+^ cells/mm^3^ in DG | | | | | |
| --- | --- | --- | --- | --- | --- |
| Genotype | Sex | n | Mean ± SEM | Two-way ANOVA | |
|  |  |  |  | Factor | Significance |
| WT | Male | 4 | 2994.0 ± 313.6 | Genotype | ** |
| WT | Female | 4 | 2792.0 ± 368.2 | Sex | ns |
| cKO | Male | 4 | 4927.0 ± 359.2 | Genotype × Sex | ns |
| cKO | Female | 4 | 4737.0 ± 206.5 |  |  |
| BrdU^+^DCX^+^ cells/mm^3^ in DG | | | | | |
| Genotype | Sex | n | Mean ± SEM | Two-way ANOVA | |
|  |  |  |  | Factor | Significance |
| WT | Male | 4 | 3803.0 ± 154.4 | Genotype | ** |
| WT | Female | 4 | 4067.0 ± 304.9 | Sex | ns |
| cKO | Male | 4 | 5673.0 ± 154.4 | Genotype × Sex | ns |
| cKO | Female | 4 | 6249.0 ± 375.1 |  |  |
| BrdU^+^NeuN^+^ cells/mm^3^ in DG | | | | | |
| Genotype | Sex | n | Mean ± SEM | Two-way ANOVA | |
|  |  |  |  | Factor | Significance |
| WT | Male | 4 | 714.4 ± 68.45 | Genotype | ** |
| WT | Female | 4 | 752.9 ± 80.56 | Sex | ns |
| cKO | Male | 4 | 1466.0 ± 148.5 | Genotype × Sex | ns |
| cKO | Female | 4 | 1236.0 ± 62.6 |  |  |
